# Locus-resolution analysis of L1 regulation and retrotransposition potential in mouse embryonic development

**DOI:** 10.1101/gr.278003.123

**Published:** 2023-09

**Authors:** Patricia Gerdes, Dorothy Chan, Mischa Lundberg, Francisco J. Sanchez-Luque, Gabriela O. Bodea, Adam D. Ewing, Geoffrey J. Faulkner, Sandra R. Richardson

**Affiliations:** 1 Mater Research Institute - University of Queensland, TRI Building, Woolloongabba, Queensland 4102, Australia;; 2 The University of Queensland Diamantina Institute, The University of Queensland, Woolloongabba, Queensland 4102, Australia;; 3 Translational Bioinformatics, Commonwealth Scientific and Industrial Research Organisation, Sydney, New South Wales 2113, Australia;; 4 GENYO. Centre for Genomics and Oncological Research (Pfizer-University of Granada-Andalusian Regional Government), PTS Granada, 18016, Spain;; 5 MRC Human Genetics Unit, Institute of Genetics and Cancer (IGC), University of Edinburgh, Western General Hospital, Edinburgh EH4 2XU, United Kingdom;; 6 Queensland Brain Institute, University of Queensland, Brisbane, Queensland 4072, Australia

## Abstract

Mice harbor ∼2800 intact copies of the retrotransposon Long Interspersed Element 1 (L1). The in vivo retrotransposition capacity of an L1 copy is defined by both its sequence integrity and epigenetic status, including DNA methylation of the monomeric units constituting young mouse L1 promoters. Locus-specific L1 methylation dynamics during development may therefore elucidate and explain spatiotemporal niches of endogenous retrotransposition but remain unresolved. Here, we interrogate the retrotransposition efficiency and epigenetic fate of source (donor) L1s, identified as mobile in vivo. We show that promoter monomer loss consistently attenuates the relative retrotransposition potential of their offspring (daughter) L1 insertions. We also observe that most donor/daughter L1 pairs are efficiently methylated upon differentiation in vivo and in vitro. We use Oxford Nanopore Technologies (ONT) long-read sequencing to resolve L1 methylation genome-wide and at individual L1 loci, revealing a distinctive “smile” pattern in methylation levels across the L1 promoter region. Using Pacific Biosciences (PacBio) SMRT sequencing of L1 5′ RACE products, we then examine DNA methylation dynamics at the mouse L1 promoter in parallel with transcription start site (TSS) distribution at locus-specific resolution. Together, our results offer a novel perspective on the interplay between epigenetic repression, L1 evolution, and genome stability.

Retrotransposons are major contributors to ongoing mutagenesis in mammalian genomes. The autonomous non-long terminal repeat (non-LTR) retrotransposon Long Interspersed Element 1 (LINE-1 or L1) is actively mobilizing in both humans and mice, and L1 sequences occupy ∼17% of human DNA and ∼18% of mouse DNA (International Human Genome Sequencing Consortium 2001; Waterston et al. 2002). While humans contain a single active L1 subfamily, the mouse genome harbors three active L1 subfamilies, termed T_F_, G_F_, and A (Wincker et al. 1987; Schichman et al. 1993; Casavant and Hardies 1994; DeBerardinis et al. 1998; Naas et al. 1998; Saxton and Martin 1998; Hardies et al. 2000; Goodier et al. 2001; Mears and Hutchison 2001) which are each further divided into several sublineages, for example, T_FI_, T_FII_, and T_FIII_ (Sookdeo et al. 2013). Although T_F_ and G_F_ elements descended from the old and now inactive F subfamilies, A elements evolved independently. However, generating a single phylogenetic tree describing the relation of the subfamilies with each other is difficult because of frequent recombination among elements (Sookdeo et al. 2013). The vast majority of the ∼600,000 L1 copies in the mouse genome reference are 5′ truncated and mutated (Voliva et al. 1983; Waterston et al. 2002), leaving approximately 2800 full-length L1s (Penzkofer et al. 2017). Ongoing L1 activity has generated substantial variation in L1 content among inbred strains, as well as interindividual variation in L1 content within strains (Akagi et al. 2008; Nellåker et al. 2012; Richardson et al. 2017; Schauer et al. 2018; Gerdes et al. 2022; Ferraj et al. 2023). L1 insertions also are responsible for several spontaneous mouse mutants, driven by L1 T_F_ elements in all cases in which the L1 subfamily can be identified (Gagnier et al. 2019).

A full-length mouse L1 is ∼6–7 kb long and begins with a 5′ untranslated region (5′ UTR) containing an internal RNA polymerase II promoter (Severynse et al. 1991; DeBerardinis and Kazazian 1999). The mouse L1 5′ UTR has a distinctive structure, wherein a variable number of tandemly repeated ∼200 bp monomer units are situated upstream of a nonmonomeric region (Adey et al. 1994; Kong et al. 2022). Each monomer contributes additive promoter activity (DeBerardinis and Kazazian 1999). Individual monomers of young L1 subfamilies generally comprise sufficient CpG dinucleotides to qualify as CpG islands (Lee et al. 2010), and also contain several transcription factor binding sites, including for YY1 transcription factor (YY1) (DeBerardinis and Kazazian 1999; Lee et al. 2010). The YY1 binding site is required for accurate L1 transcription initiation (Athanikar et al. 2004; Lee et al. 2010), and an intact YY1 binding site also is important for methylation of the human L1 promoter during cellular differentiation (Sanchez-Luque et al. 2019).

The L1 5′ UTR is followed by two open reading frames encoding the proteins ORF1p and ORF2p, and a 3′ UTR incorporating a polyadenylation signal (Scott et al. 1987; Skowronski et al. 1988; Dombroski et al. 1991). ORF1p is ∼40 kD and harbors RNA binding and chaperone activities (Holmes et al. 1992; Hohjoh and Singer 1996; Martin and Bushman 2001; Khazina and Weichenrieder 2009, 2018) whereas the ∼150 kD ORF2p has showed endonuclease (EN) and reverse transcriptase (RT) activities (Mathias et al. 1991; Feng et al. 1996; Ergün et al. 2004; Doucet et al. 2010; Taylor et al. 2013). Both proteins are required for L1 mobilization through reverse transcription of an RNA intermediate in a process termed target-site primed reverse transcription (TPRT) (Scott et al. 1987; Holmes et al. 1992; Luan et al. 1993; Feng et al. 1996; Moran et al. 1996). While L1 retrotransposition can occur in nondividing cells (Kubo et al. 2006; Macia et al. 2017), a growing body of evidence has emerged linking L1 retrotransposition to DNA replication during the S phase of the cell cycle (Mita et al. 2018, 2020; Flasch et al. 2019). Hallmarks of L1 integration by TPRT include flanking target site duplications (TSDs), and the incorporation of a 3′ poly(A) tract which reflects the necessity of L1 mRNA polyadenylation for efficient retrotransposition (Grimaldi et al. 1984; Doucet et al. 2015).

Unchecked retrotransposition presents a threat to genome stability, and is countered by a variety of host defense mechanisms (Bourc'his and Bestor 2004; Goodier 2016; MacLennan et al. 2017; Liu et al. 2018; Deniz et al. 2019; Greenberg and Bourc'his 2019; Mita et al. 2020; Tristán-Ramos et al. 2020; Senft and Macfarlan 2021). While repressive histone marks such as H3K9me3 play a major role in silencing older mouse L1 subfamilies (Tan et al. 2013; Castro-Diaz et al. 2014; Jacobs et al. 2014), younger L1s are typically silenced by methylation of the CpG islands in their promoters (Furano et al. 1988; Hata and Sakaki 1997; Lee et al. 2010; de la Rica et al. 2016; Gerdes et al. 2022). During embryonic development, the epigenome undergoes reprogramming including phases of global DNA demethylation (Hajkova et al. 2002; Seki et al. 2005; Abe et al. 2011; Saitou et al. 2012; Seisenberger et al. 2012; Smith et al. 2012; Cantone and Fisher 2013). The developmental methylation dynamics of L1 promoters have been examined using subfamily-specific and whole genome bisulfite sequencing (WGBS) (Hajkova et al. 2002; Kuramochi-Miyagawa et al. 2008; Watanabe et al. 2008; Popp et al. 2010; Saitou et al. 2012; Seisenberger et al. 2012; Smith et al. 2012; Molaro et al. 2014; Schöpp et al. 2020; Zoch et al. 2020). However, because of the repetitive structure and variable length of the mouse L1 promoter, as well as the high sequence identity among young L1 copies in the genome, assignment of short internal reads to specific mouse L1 loci is challenging (Lanciano and Cristofari 2020), and assessment of L1 methylation status *en masse* may mask individual L1s whose methylation dynamics differ from those of their subfamily. Indeed, studies of human L1s suggest certain loci can “escape” methylation and thus contribute to somatic retrotransposition throughout development and in cancer (Pitkänen et al. 2014; Tubio et al. 2014; Paterson et al. 2015; Philippe et al. 2016; Scott et al. 2016; Gardner et al. 2017; Nguyen et al. 2018; Schauer et al. 2018; Salvador-Palomeque et al. 2019; Sanchez-Luque et al. 2019; Ewing et al. 2020). Locus-specific resolution of murine developmental L1 methylation, however, remains largely unexplored. Heritability of locus-specific retrotransposon methylation has been found in the context of “metastable epialleles” mostly involving variably methylated young IAP elements (VM-IAPs) (Bertozzi and Ferguson-Smith 2020). However, genome-wide screens have not revealed evidence of this phenomenon for L1s (Kazachenka et al. 2018; Elmer et al. 2021).

We previously characterized five de novo (daughter) L1 insertions arising in pedigrees of inbred mice (Richardson et al. 2017), and identified their source (donor) elements through unique L1 3′ transductions (Holmes et al. 1994; Moran et al. 1999; Goodier et al. 2000; Pickeral et al. 2000; Xing et al. 2006; Beck et al. 2010). In this study, we use the mosaic tissues from the animals in which these insertions arose and their heterozygous insertion-bearing offspring, to investigate the retrotransposition potential and epigenetic fate of de novo and retrotransposition-competent L1 copies in vivo. We also use in vitro differentiation of mouse embryonic stem cells (mESCs) as a model to explore developmental L1 methylation dynamics at locus-specific resolution and genome-wide. We use cell culture-based L1 retrotransposition assays, locus-specific bisulfite sequencing, Oxford Nanopore Technologies (ONT) long-read DNA sequencing and methylation profiling, and Pacific Biosciences (PacBio) SMRT sequencing of L1 5′ RACE cDNAs to explore the relationship between developmental DNA methylation and L1 expression and retrotransposition capacity at single-locus resolution.

## Results

### L1 retrotransposition efficiency is diminished by ongoing promoter shortening

**Figure 1.**
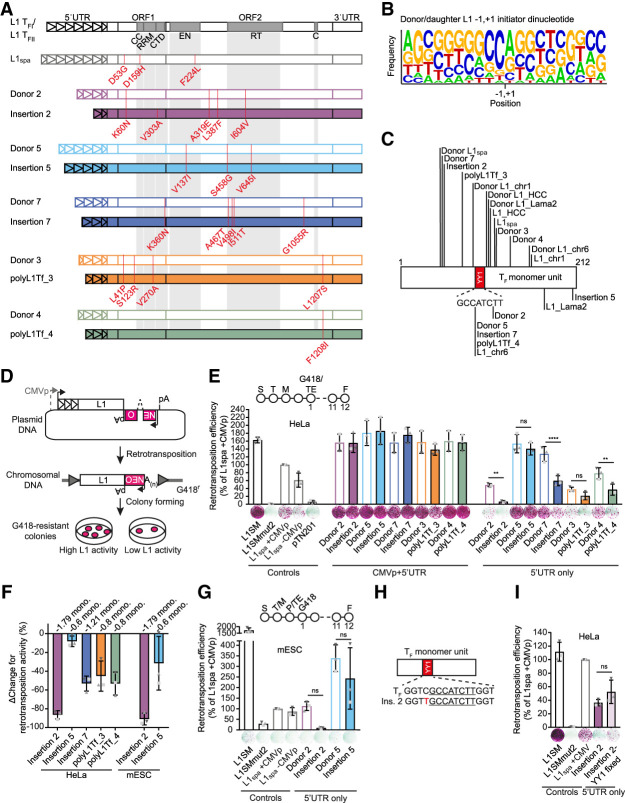
L1 donor/daughter pairs retrotranspose efficiently in vitro. (*A*) Amino acid changes in ORF1 and ORF2 compared to the L1 T_FI_ and L1 T_FII_ consensus sequences (Sookdeo et al. 2013) are annotated in red. L1_spa_ refers to the published disease-causing insertion used in later experiments (Naas et al. 1998). Functional domains in ORF1 and ORF2 are shown: CC = coiled-coiled, RRM = RNA recognition motif, CTD = C-terminal domain, EN = endonuclease, RT = reverse transcriptase, C = cysteine-rich motif. Triangles within the 5′ UTR represent monomer units. For nucleotide substitutions in promoters see Supplemental Figure S1. (*B*) Sequence logo (Crooks et al. 2004) of putative transcription initiation start sites for all ten donor and daughter L1 pairs. Sequence represents transcription initiator dinucleotide in the center ± 9 nucleotides upstream and downstream. −1,+1 indicates transcription initiator dinucleotide. The first nucleotide of L1 sequence corresponds to the second nucleotide in transcription initiator dinucleotide. The position of the first base of each daughter L1 relative to its donor element, and the first base of each donor L1 relative to the L1 T_FI_/T_FII_ consensus sequences was analyzed. (*C*) Donor and daughter L1 5′ truncation points. Sequences or locations of donors and daughters were previously published as indicated in Table 1 (Kingsmore et al. 1994; Naas et al. 1998; Besse et al. 2003; Richardson et al. 2017; Schauer et al. 2018; Gagnier et al. 2019). Lines indicate truncation points of elements in the 5′-most monomer. YY1 binding site (GCCATCTT) is shown in red. (*D*) Rationale of a cultured cell retrotransposition assay (Moran et al. 1996; Wei et al. 2000). Constructs used in this study were previously published (Moran et al. 1996; Goodier et al. 2001; Han and Boeke 2004) or generated by modifying the pTN201 construct [L1_spa_ (Naas et al. 1998)]. An antisense orientated neomycin-resistance (NEO^r^) reporter cassette interrupted by a sense-oriented intron is inserted into a mouse L1 3′ UTR. The mouse L1 is driven by its native 5′ UTR promoter or a CMV promoter (CMVp). Cells harboring a retrotransposition event become neomycin (G418) resistant. The colony number reflects the relative activity of the L1 construct. (*E*) Comparison of L1 donor/daughter pair retrotransposition efficiency in HeLa cells. The retrotransposition assay timeline is shown in the *top* (S: seeding, T: transfection, M: change of media, G418: start of G418 selection, TE: measurement of transfection efficiency, F: Fixing and staining of colonies). Constructs: L1SM (positive control), L1SMmut2 (negative control), pTN201, L1_spa_ +CMVp/−CMVp, L1 donor/daughter pairs +CMVp/−CMVp. Colony counts were normalized to L1_spa_ + CMVp and are shown as mean ± SD of three independent biological replicates, each of which comprised three technical replicates. (*) *P* ≤ 0.0332, (**) *P* ≤ 0.0021, (***) *P* ≤ 0.0002, (****) *P* < 0.0001, ns = not significant (One-way ANOVA followed by Sidak's post-hoc test, *P* = 0.0060, 0.7632, <0.0001, 0.5652, 0.0066 for donor/daughter pairs –CMVp from *left* to *right*). Representative well pictures are shown *below* each construct. 5 × 10^3^ cells were plated per well in a six-well plate. (*F*) Percentage change (ΔChange) in retrotransposition activity between L1 donor/daughter pairs. Shown is the decrease of retrotransposition efficiency per daughter L1 compared to its respective donor L1. Data is shown as mean ± SD of three independent biological replicates. (*G*) Comparison of L1 donor/daughter pair retrotransposition efficiency in mESCs. The retrotransposition assay timeline is shown at the *top* (S: seeding, T: transfection, M: change of media 8 h after transfection, P: passaging of cells into 10 cm plates, TE: measurement of transfection efficiency, G418: start of G418 selection, F: Fixing and staining of colonies). Constructs as described in (*E*). Colony counts were normalized to L1_spa_ + CMVp and are shown as mean ± SD of three independent biological replicates, each of which comprised two technical replicates. (*) *P* ≤ 0.0332, (**) *P* ≤ 0.0021, (***) *P* ≤ 0.0002, (****) *P* < 0.0001, ns = not significant (One-way ANOVA followed by Sidak's post-hoc test, *P* = 0.2851, 0.3305 for donor/daughter pairs –CMVp from *left* to *right*). Representative well pictures are shown *below* each construct. 4 × 10^5^ cells were plated per well in a six-well plate. (*H*) Schematic of an L1 monomer unit. The YY1 binding site is indicated as red rectangle. The extended YY1 binding motif sequence is shown *below*. The core YY1 binding motif sequence is underlined. A mutation in the extended YY1 binding motif sequence adjacent to the core motif in Insertion 2 is indicated in red. (*I*) Comparison of retrotransposition efficiency of Insertion 2 and Insertion 2 with intact YY1 binding sites (Insertion 2-YY1 fixed) in retrotransposition assay in HeLa cells. Constructs as described in (*D*). Colony counts were normalized to L1_spa_ in pCEP4-mneoI-G4 + CMVp and are shown as mean ± SD of three independent biological replicates, each of which comprised three technical replicates. (*) *P* ≤ 0.05, ns = not significant (two-tailed *t*-test, *P* = 0.1607). Representative well pictures are shown *below* each construct. 1 × 10^4^ cells were plated per well in a six-well plate. Note: L1SM retrotransposed very efficiently, leading to cell colony crowding in wells, and a likely underestimate of retrotransposition.

To evaluate the retrotransposition potential of de novo and polymorphic daughter elements relative to their donors, we amplified via polymerase chain reaction (PCR), cloned and capillary sequenced five donor/daughter pairs (Richardson et al. 2017) to derive the exact nucleotide sequence of each element (Sanchez-Luque et al. 2019) (see Methods). All 10 L1s contained at least one T_F_ monomer unit and encoded intact open reading frames (ORFs), and each daughter L1 contained between 0.6 and 1.8 fewer monomer units than the corresponding donor L1 (Table 1; Fig. 1A; Supplemental Fig. S1A). The remaining sequence of each daughter L1 was identical to its donor, with the exception of Insertion 2 which had a single nonsynonymous substitution in ORF1 (V303A) (Fig. 1A; Supplemental Fig. S1A). This result, representing a single nucleotide substitution among 33,374 reverse transcribed bases, is consistent with a high fidelity for mouse L1 reverse transcriptase activity in vivo.

**Table 1.**
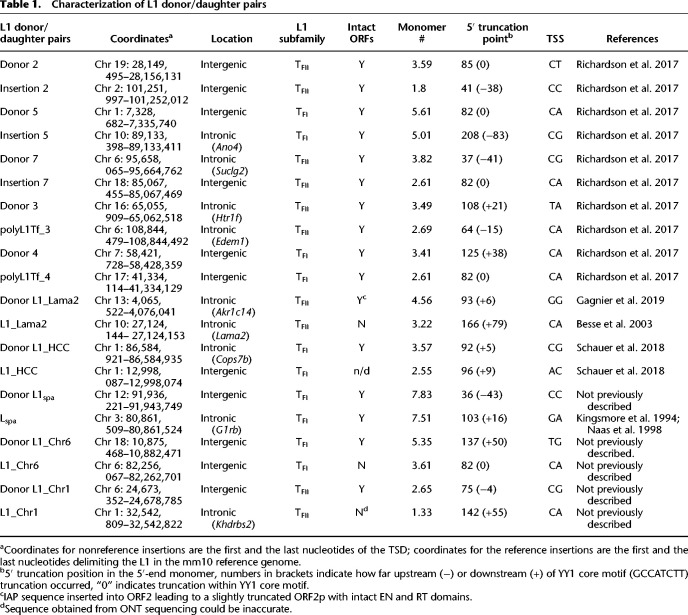
Characterization of L1 donor/daughter pairs

The loss of daughter element 5′ UTR sequence could be explained either by 5′ truncation during retrotransposition (Ostertag and Kazazian 2001; Symer et al. 2002; Zingler et al. 2005), or by the use of TSSs within internal monomers of the donor element promoter (DeBerardinis and Kazazian 1999). We analyzed the putative initiator dinucleotide (−1,+1) (Carninci et al. 2006) for the 10 elements under study (Fig. 1A), as well as 10 additional L1 T_F_ elements (Supplemental Fig. S1B) comprising five likely donor/daughter pairs (Table 1). Under the assumption that the most 5′ position of each element represents the first transcribed nucleotide, transcription of 14/20 elements initiated at the preferred mammalian PolII initiator pyrimidine/purine dinucleotide (Carninci et al. 2006) (Fig. 1B; Supplemental Fig. S1C; Table 1). 15 of the 20 elements analyzed here were 5′ truncated within the first 108 nt of the 5′-most T_F_ monomer. 5/20 elements truncated within, and an additional 7/20 truncated in close proximity (≤21 bp) to, the YY1 core binding motif (GCCATCTT) (Fig. 1C; Table 1) as previously observed for mouse L1s (Shehee et al. 1987; DeBerardinis and Kazazian 1999; Zhou and Smith 2019). The observed clustering of 5′ truncation points, and their coincidence with the Py/Pu initiator dinucleotide, are consistent with the daughter L1 promoters being shortened due to transcription initiation internal to the 5′ UTR of the donor element.

To quantify the impact of monomer loss on daughter element mobility, we evaluated the five donor/daughter pairs identified in our previous study (Richardson et al. 2017) in a cultured cell L1 retrotransposition assay (Moran et al. 1996; Wei et al. 2000). We tested each element driven either by a cytomegalovirus promoter and the native L1 promoter (CMVp + 5′ UTR), or by the native L1 promoter only (5′ UTR only), and quantified their activity relative to L1_spa_ (Fig. 1D; Kingsmore et al. 1994; Naas et al. 1998). In HeLa cells, when driven by CMVp + 5′ UTR, all elements mobilized efficiently (∼160% of L1_spa_), with donor and daughter elements showing similar activity. Notably, Insertion 2 which has an amino acid change in ORF1p retrotransposed with the same efficiency as its donor (∼160%) when transcribed from the CMVp, indicating that the mutation does not influence retrotransposition efficiency. In contrast, when driven by the 5′ UTR alone, each daughter element mobilized less efficiently than its donor (Fig. 1E,F). This trend was most pronounced for Insertion 2, which retrotransposed at 8% of L1_spa_ + CMVp, compared to 62% for Donor 2, and reached statistical significance for three of the five donor/daughter pairs (One-way ANOVA followed by Sidak's post-hoc test, *P* = 0.0060, 0.7632, <0.0001, 0.5652, 0.0066 for donor/daughter pairs 2, 5, 7, 3 and 4, respectively; Fig. 1E,F). A similar trend was observed when donor/daughter pairs 2 and 5 (5′ UTR only) were tested in mESCs (MacLennan et al. 2017), but did not reach statistical significance for either pair (One-way ANOVA followed by Sidak's post-hoc test, *P* = 0.2851, 0.3305 for pairs 2 and 5, respectively; Fig. 1F,G). We also noted that Insertion 2 had a single nucleotide mutation (1115C > T) in the extended YY1 binding motif (GGTCGCCATCTTGGT) in its second monomer (Fig. 1H) which could decrease YY1 binding affinity (Kim and Kim 2009). To determine whether the 1115C > T mutation impacts the retrotransposition efficiency of this element, we restored the YY1 binding site (Insertion 2-YY1 fixed), and observed a ∼15% increase in retrotransposition activity which was not statistically significant (two-tailed *t*-test, *P* = 0.1607; Fig. 1I). Together, these results are consistent with previous luciferase reporter assays showing mouse L1 promoter strength is proportionate to monomer number (DeBerardinis and Kazazian 1999). Moreover, we find that monomer loss consistently diminishes the retrotransposition potential of de novo mouse L1 insertions relative to their donor elements.

### Donor and daughter L1 insertions are largely methylated in adult tissues

**Figure 2.**
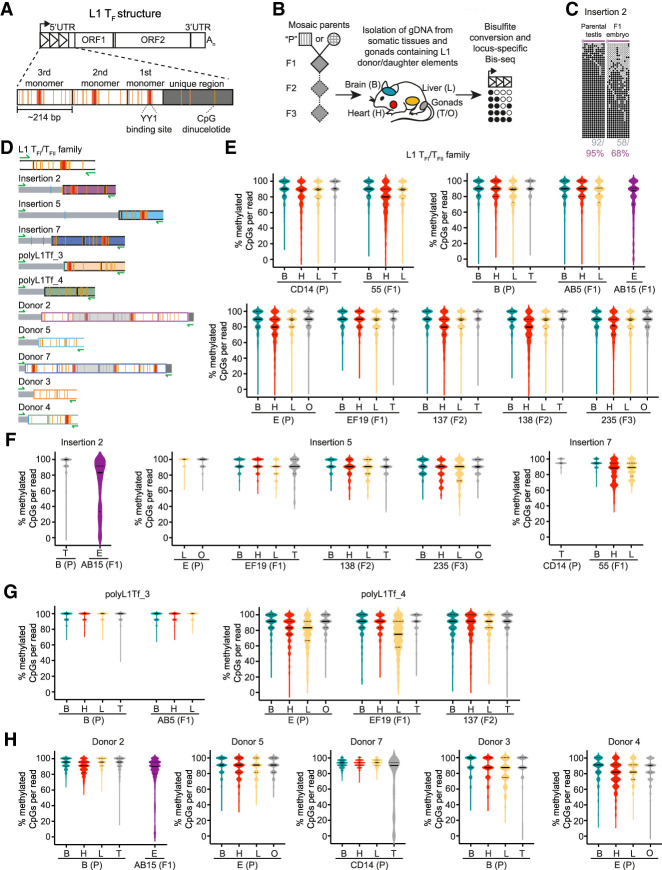
L1 donor/daughter elements are methylated in somatic tissues of adult mice. (*A*) Schematic of CpG dinucleotides in a mouse L1 T_F_ element. Triangles in 5′ UTR represent monomer units. A magnification of the 5′ UTR is shown *below*. Black boxes represent monomer units. Dark gray box represents unique (nonmonomeric) region within 5′ UTR. Orange strokes represent CpG dinucleotides. Red boxes represent YY1 binding sites. (*B*) Experimental design of mouse L1 locus-specific bisulfite sequencing. Genomic DNA was extracted from tissues of C57BL6/J mice harboring previously identified donor and daughter L1 insertions (Richardson et al. 2017). The parental generation “P” (square = male, circle = female) is mosaic for the de novo daughter L1 insertion (represented by stripes). F1-F3 generations are heterozygous for L1 insertions (filled diamond = male or female). F2 and F3 generation animals were only available for Insertion 5 and polyL1Tf_4. Tissues for analysis of donor elements were only collected from the P generation. DNA was isolated from brain (green), heart (red), liver (orange), and gonads (gray) if available. After bisulfite conversion, the 5′ monomeric region of each L1 was PCR amplified. Amplicons were pooled and sequenced as 2 × 300-mer Illumina reads. Circles represent methylated (black circles) and unmethylated (white circles) CpG dinucleotides in L1 5′ UTR. (*C*) Methylation of a de novo L1 promoter sequence (Insertion 2) shown in the germline mosaic parental testis (animal B) and in the following F1 generation embryonic tissue (animal AB15). Displayed are 50 nonidentical sequences extracted at random from a much larger pool of available Illumina reads. Each cartoon panel corresponds to an amplicon (black circle, methylated CpG; white circle, unmethylated CpG; ×, mutated CpG). Colored line *above* the cartoon represents amplicon (gray = genomic sequence, colored = L1 sequence). The overall percentage of methylated CpG dinucleotides is indicated *below* each cartoon. Gray letters indicate methylation of CpG dinucleotides in genomic sequence. Colored letters indicate methylation of CpG dinucleotides in L1 sequence. (*D*) Locus-specific methylation analysis schematic representation for L1 T_F_ monomer, 3 full-length de novo L1 insertions (Insertion 2, 5, 7), 2 polymorphic L1 insertions (polyL1Tf_3 and polyL1Tf_4) and their 5 respective donor elements (Donor 2, 5, 7, 3, 4). 5′ monomeric sequences of each L1 were PCR amplified using primer pairs (green arrows) specific to that locus. Orange strokes indicate L1 CpG dinucleotides covered by the assay. Blue strokes represent covered genomic CpG dinucleotides. Gray strokes in the gray shaded area represent CpG dinucleotides not reached by Illumina sequencing. Red boxes indicate YY1 binding sites. Colored boxes represent L1 monomer units. (*E*) Genome-wide methylation of L1 T_FI_/T_FII_ promoter sequence shown in all animals. Animals are labeled E, EF19, 137, 138, 235, B, AB5, AB15, CD14 and 55 in the *bottom* part of the *x*-axis label, with the generation (P, F1, F2 or F3) indicated in brackets. Each graph contains animals from the same family. The different tissues used for DNA extraction and bisulfite sequencing in each animal is indicated in the *top* part of the *x-*axis labeled as: brain, B; heart, H; liver, L; testis, T; ovaries, O; and embryonic tissues, E. Displayed are 1000 nonidentical sequences extracted at random from a much larger pool of available Illumina reads. The violin plots represent the methylation distribution as per Supplemental Figure S2. The black line and dashed lines show the distribution median and quartiles, respectively. The percentage of methylated CpGs per read is indicated on the *y*-axis. (*F*) As for (*E*) but for de novo L1 promoter sequences shown in the mosaic P generation in which each de novo L1 insertion was identified (animals E and CD14) and in following F1–F3 generations if available (animals EF19, 138, 235 and 55). Displayed are 1000 nonidentical sequences (if available) extracted at random from a much larger pool of available Illumina reads (exceptions: Insertion 5: 138 H, 235 B, H, L, O, EF19 T [368, 328, 337, 352, 958, 282 reads, respectively]; Insertion 7: 55 B, H, L, CD14 T [598, 885, 890, 662 reads, respectively]). (*G*) As for (*E*,*F*) but for polymorphic L1 insertions. (*H*) As for (*E*,*F*) but showing methylation of 5 donor L1 elements in the animal they mobilized in (P generation).

Having established the retrotransposition competence of the donor and daughter elements in vitro, we next used mouse L1 locus-specific bisulfite sequencing (Schauer et al. 2018) to evaluate the methylation status of each L1 in the somatic tissues and gonads of the mosaic animal in which the daughter insertion arose, and in subsequent generations of heterozygous insertion-bearing animals (Fig. 2A–D). For comparison, we analyzed the genome-wide methylation of the T_FI_ and T_FII_ subfamily monomers using primers internal to the monomer sequence (Schauer et al. 2018). Because of their high sequence similarity, it was not possible to design primers specific to the T_FI_ or T_FII_ subfamily. Overall, the T_FI_/T_FII_ subfamily monomer sequence was >80% methylated in adult tissues, although a few demethylated reads were observed across animals and tissues (Fig. 2E; Supplemental Fig. S2A).

The three de novo daughter L1s (Insertions 2, 5, and 7) (Table 1) were >80% methylated in the somatic tissues and gonads of adult mice, including mosaic animals E (Insertion 5), CD14 (Insertion 7), and their heterozygous F1, F2, and F3 descendants (Fig. 2C,F; Supplemental Fig. S2B). Notably, Insertion 2 originated as germline-restricted mosaic in mouse B and was transmitted only to F1 animal AB15, which was harvested as a postimplantation embryo. Insertion 2 was highly methylated in the adult testis of mouse B, but partially demethylated in embryo AB15 (Fig. 2C,F). As the genomic DNA of embryo AB15 was analyzed in bulk, the demethylated sequences may potentially correspond to primordial germ cells (PGCs) or multipotent stem cells. Consistently, the T_FI_/T_FII_ subfamily monomer sequence and Donor 2 (see below) also showed partial demethylation in embryo AB15 (Fig. 2E). We also analyzed the methylation status of unfixed polymorphic insertions polyL1Tf_3 and polyL1Tf_4 (Table 1). Insertion polyL1Tf_3 was nearly 100% methylated across all adult tissues examined (Fig. 2G; Supplemental Fig. S2B). However, methylation of polyL1Tf_4 was more relaxed (<90%), with a tendency to be especially demethylated in liver (Fig. 2G; Supplemental Fig. S2B). This variability may reflect the influence of genomic location and physiological context on L1 element methylation (Salvador-Palomeque et al. 2019; Sanchez-Luque et al. 2019; Ewing et al. 2020). Together, these results indicate that de novo L1 insertions arising during embryonic development are likely silenced by DNA methylation during later embryogenesis. This methylation is maintained in subsequent generations, with an average methylation level of 93% in brain, 89% in heart and 86% in liver for daughter insertions and 91% in brain, 87% in heart and 88% in liver for the donor L1s.

We viewed donor L1s active during embryonic development as candidate “escapee” loci in mice, potentially akin to specific human L1 loci that evade epigenetic repression in differentiated cells (Scott et al. 2016; Salvador-Palomeque et al. 2019; Sanchez-Luque et al. 2019; Ewing et al. 2020). We therefore assessed methylation of Donor 2, Donor 5, and Donor 7 in somatic tissues and germ cells of their mosaic founder animals (mouse B, mouse E, and mouse CD14, respectively). We also analyzed methylation of Donor 3 and Donor 4 in animals that carried the respective polymorphic daughter insertions polyL1Tf_3 and polyL1Tf_4 (Fig. 2B,D). Nearly all donor elements showed >80% methylation in somatic tissues and gonads (Fig. 2H; Supplemental Fig. S2C). The exception was Donor 7 which, although it was completely methylated in the somatic tissues of founder mouse CD14, was hypomethylated in the germ cell fraction of mouse CD14 testis. This is a notable departure from the genome-wide state of T_FI_/T_FII_ monomer sequences, which we found to be largely methylated in adult gonads (Fig. 2E,H; Supplemental Fig. S2A,C). Thus, Donor 7 may represent an L1 that is refractory to methylation during germline development and therefore privileged for heritable retrotransposition.

### Young L1s are rapidly methylated during in vitro mESC differentiation

**Figure 3.**
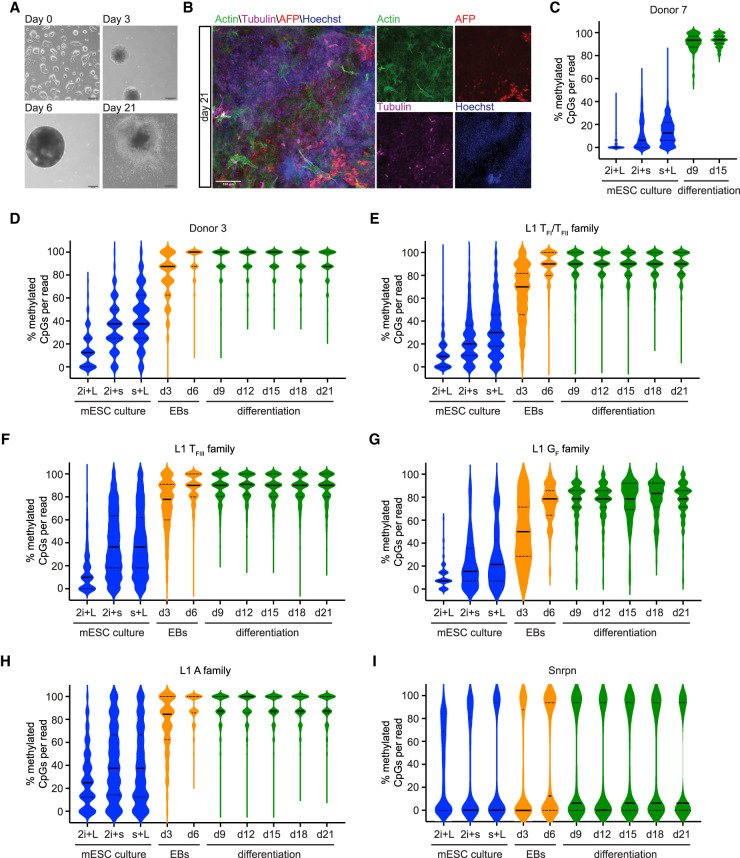
Dynamic methylation of L1 elements during differentiation of mESCs. (*A*) Differentiation of mESCs to cells of all three germ layers using a standard differentiation protocol (Behringer et al. 2016). Undifferentiated E14 mESCs are grown on gelatin (day 0). Embryoid bodies (EBs) are generated by “hanging drop culture” (day 3) and are grown in suspension culture (day 6). After 6 d, EBs are plated and differentiated for 2 wk (day 21). Scale bar, 200 µm. (*B*) Immunofluorescence image of mesodermal (Actin, green), endodermal (AFP, red) and ectodermal (Tubulin, violet) lineage markers in differentiated E14 mESCs on day 21. Nuclei were stained with Hoechst (blue). Scale bar, 100 µm. (*C*) Methylation of Donor 7 promoter sequence shown in the mESCs cultured in three different conditions (2i + L = 2i + LIF, 2i + s = 2i + serum, s + L = serum + LIF) and on differentiation day 9 (d9) and day 15 (d15). Because of the technical challenge posed by PCR amplification of long bisulfite-treated fragments, sufficient material was generated to assess Donor 7 methylation only at day 9 and day 15 of differentiation. Displayed are 1000 nonidentical sequences (if available) extracted at random from a much larger pool of available Illumina reads (exceptions: Donor 7: 2i + LIF, 2i + serum, serum + LIF, d9, d15 [874, 837, 876, 110, 124 reads, respectively]). The violin plots represent the methylation distribution as per Supplemental Figure S4. The black line and dashed lines show the distribution median and quartiles, respectively. (*D*–*I*) As for (*C*) but for Donor 3 (*D*), L1 T_FI_/ T_FII_ family (*E*), L1 T_FIII_ family (*F*), L1 G_F_ family (*G*), L1 A family (*H*), and the imprinted gene *Snrpn* (*I*). Primers for L1 families are within the L1 promoter sequence. Shown is methylation in three different mESC culture conditions, during EB culture and during differentiation. Displayed are 1000 nonidentical sequences (if available) extracted at random from a much larger pool of available Illumina reads (exceptions: L1 G_F_ family: 2i + LIF, 2i + serum, serum + LIF, d3, d6, d9, d12, d15, d18, d21 [86, 127, 119, 120, 168, 162, 161, 155, 155, 175 reads, respectively]).

As adult tissues reveal only the end point of developmental L1 methylation dynamics, we next analyzed L1 methylation at genome-wide and locus-specific resolution during cellular differentiation. To model various states of pluripotency, we cultured feeder-free E14 mESCs in three conditions: serum complemented with leukemia inhibitory factor (serum + LIF), which generates a heterogeneous population of mESCs in a pluripotent state (Smith et al. 1988; Williams et al. 1988); two small kinase inhibitors + LIF (2i + LIF), which maintains the mESCs in a naive ground state similar to that of the inner cell mass (ICM) (Silva et al. 2008); and under 2i + serum conditions, which are shown to support engineered mouse L1 retrotransposition (MacLennan et al. 2017). To recapitulate specification and differentiation of cells into the three germ lineages (ectoderm, mesoderm, and endoderm), we collected genomic DNA over a time course, from differentiation induction of serum + LIF mESCs to embryoid bodies (EBs) for 6 d, and through subsequent differentiation over 15 d (Fig. 3A,B; Supplemental Fig. S3A,B; Behringer et al. 2016). L1 methylation state was assessed using genome-wide and locus-specific L1 bisulfite sequencing in the three mESC culture conditions and on days 3, 6, 9, 12, 15, 18 and 21 of differentiation.

The mobile L1 subfamilies T_F_, G_F_ and A each showed their lowest methylation levels in 2i + LIF ground-state conditions, and reached maximal methylation by day 6 of differentiation (Fig. 3E–H; Supplemental Fig. S4C–F). Notably, the G_F_ subfamily was less methylated (<80%) than the other subfamilies (>80%) both during differentiation and in fully differentiated EBs (Fig. 3G; Supplemental Fig. S4F). The 129/Ola genetic background from which E14 mESCs are derived contained two of the donor L1s analyzed above, Donor 3 and Donor 7. Both loci were largely demethylated (<50%) across all mESC culture conditions (Fig. 3C,D; Supplemental Fig. S4A,B). Donor 3 showed 80% methylation at day 3 of EB differentiation, and >90% at day 6. Donor 7 showed >90% methylation at day 9 and day 21 (Fig. 3C; Supplemental Fig. S4A). These results were in line with our observation that both donors were completely methylated in somatic tissues of adult mice (Fig. 2H) and is consistent with methylation occurring during differentiation in embryonic development in vivo. As an internal control, analysis of the maternally imprinted *Snrpn* gene revealed the expected bimodal distribution of methylation in pluripotent cells and across the differentiation time course (Fig. 3I; Supplemental Fig. S4G). Together, these results show rapid remethylation of L1 sequences during cellular differentiation, with subtle but notable variability between active L1 subfamilies and among individual loci.

### Methylation fluctuates within mouse L1 promoters

**Figure 4.**
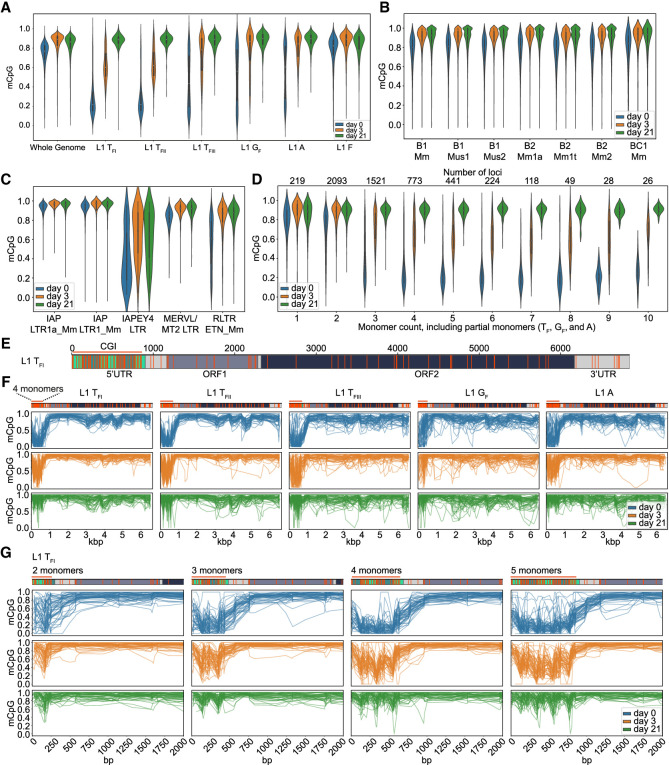
ONT CpG methylation profiles of TEs. (*A*) Violin plots are showing methylated CpG fraction for the whole genome (6 kbp windows), L1 5′ UTRs belonging to the active L1 T_FI_, T_FII_, T_FIII_, G_F_ and A subfamilies and the evolutionary older and inactive L1 F subfamily at three time points of differentiation: d0 (undifferentiated mESCs in serum + LIF), d3 (EBs on day 3 of differentiation) and on d21 (completely differentiated cells). (*B*) As for (*A*), but for B1, B2 and BC1 SINE subfamilies. (*C*) As for (*A*), but for IAP LTR1a_Mm and LTR1_Mm, IAPEY4 LTR, MERV-L/MT2 LTR and RLTR ETn_Mm copies. (*D*) As for (*A*), but for violin plots showing the methylated CpG fraction of active L1 subfamily (T_F_, G_F_, and A together) promoters (monomers only) depending on the number of monomers (including partial monomers). Only elements with a minimum coverage of five reads across the whole 5′ UTR were included in the plot. The number of loci represented in each bin is shown in the *top*. (*E*) Annotated full-length L1 T_F1_ consensus showing the monomer units in green, unique region in light gray, ORF1 in dark gray, ORF2 in dark green, and 3′ UTR in light gray. CpG dinucleotides throughout the whole element are displayed as orange strokes. The promoter CpG island (CGI) is indicated as an orange line. Number of bp are shown *above* the element. (*F*) Data is shown for full-length L1s (T_FI_, T_FII_, T_FIII_, G_F_ and A) containing four monomers in the promoter at three time points of differentiation: d0 (undifferentiated mESCs in serum + LIF), d3 (EBs on day 3 of differentiation), and on d21 (completely differentiated cells). Each graph displays up to 50 methylation profiles for the specified L1 subfamily. Annotated consensus sequences as per (*E*) are shown at *top* including CpG positions. (*G*) As for (*F*), but for promoters of L1 T_FI_ subfamily members containing between 2 and 5 monomers at three time points of differentiation (d0, d3, and d21).

Although locus-specific bisulfite sequencing allows base-pair resolution of individual L1 methylation, it is limited to the 5′-most portion of each L1 promoter. To attain complete methylation profiles of L1 loci without bisulfite conversion, we performed PCR-free ONT sequencing of mESCs in serum + LIF (day 0; d0), day 3 (d3) EBs, and day 21 (d21) differentiated cells. We achieved ∼15–20× genome-wide depth using an ONT PromethION platform, and surveyed CpG methylation via the methylartist package (Ewing et al. 2020; Cheetham et al. 2022). Evaluated by ONT sequencing, DNA methylation increased during differentiation (Fig. 4A). Examining the methylation profiles of each L1 subfamily averaged across their 5′ UTRs, we found that in d0 mESCs the 5′ UTRs of full-length T_F_, G_F_ and A subfamily L1s were less methylated compared to the older L1 F subfamily (Fig. 4A). Average methylation of all L1 subfamilies rapidly increased to ∼80% by day 3 of differentiation; however, T_FI_ and T_FII_ elements were less methylated (≤80%) in d3 EBs compared to other L1 subfamilies (Fig. 4A). B1, B2, and BC1 SINEs were only slightly demethylated in d0 mESCs (∼80%), and fully methylated (>90%) in d3 EBs and d21 fully differentiated cells (Fig. 4B). While IAP retrotransposon LTRs were generally almost 100% methylated at all three differentiation time points, the IAP subfamily IAPEY4 LTRs showed an average methylation level of <40% in d0 mESCs and only ∼70% methylation in d3 EBs and d21 differentiated cells. MERVL/MT2 LTRs were >80% methylated at all three time points. Etn elements showed an average methylation level of only ∼60% in d0 mESCs but >80% in d3 EBs and d21 differentiated cells (Fig. 4C).

Next, we quantified the methylation of active L1 subfamily promoters independently of the L1 body (unique 5′ UTR region, ORFs, 3′ UTR). We binned promoters genome-wide based on monomer count, with the majority of young L1 subfamily members containing between 2 and 5 monomer units (Fig. 4D), consistent with previous analyses of the mouse reference genome (Zhou and Smith 2019). On the whole, L1 promoter methylation was distributed between ∼0% and 90% in the d0 mESCs, indicating variability among loci even in pluripotent serum + LIF culture conditions. Methylation of L1 loci in d3 EBs was higher than in d0 mESCs but a substantial proportion of L1s were still <80% methylated. Notably, none of the L1 loci displayed here appeared to be completely demethylated in d3 EBs. Methylation of the majority of L1s was re-established (>80%) in d21 differentiated cells (Fig. 4D). However, some loci appeared to be <70% methylated even in d21 differentiated cells. We examined three of these methylation “escapees” and found for each a mixture of methylated and demethylated reads at d21 potentially belonging to specific cell types in our mixed population of differentiated cells (Supplemental Fig. S5) indicating again a likely lineage or cell type specificity for L1 methylation “escapees” (Salvador-Palomeque et al. 2019; Sanchez-Luque et al. 2019; Ewing et al. 2020). Based on the mouse genome reference sequence all three L1s contain intact ORFs and multiple (4–7) monomers, indicating their potential retrotransposition competence.

We next assessed composite methylation profiles covering the previously inaccessible interiors of full-length mouse L1s and in particular the entire mouse L1 promoter (Fig. 4E). We observed a consistent methylation trough in the 5′ UTR promoter region in d0 mESCs and d3 EBs, whereas the L1 body was consistently hypermethylated at all three time points (Fig. 4F). Zooming in on the promoter region of the composite L1 methylation profiles revealed a consistent “smile” pattern across the 5′ UTR, with the innermost monomers less methylated compared to the 5′-most and 3′-most monomers (Fig. 4G; Supplemental Fig. S6). This methylation pattern was most pronounced in T_FI_ and T_FII_ elements, and in elements containing three or more monomer units (Fig. 4G; Supplemental Fig. S6A–D). Elements with two monomers appeared highly variable in their methylation status in d0 mESCs (Fig. 4G; Supplemental Fig. S6A–D). Our analyses also revealed peaks and valleys of methylation along mouse L1 promoters, with a periodicity corresponding to the monomer units. Taking advantage of the locus-specific resolution offered by ONT sequencing, we examined methylation of the two donor L1s (Donor 3 and Donor 7) present in E14 mESCs (Supplemental Fig. S7). These elements recapitulated the methylation trough observed in the composite plots in d0 mESCs and d3 EBs. As an internal control, we readily identified the differentially methylated regions (DMRs) of two imprinted genes, *Snrpn* and *Impact* (Supplemental Fig. S8).

**Figure 5.**
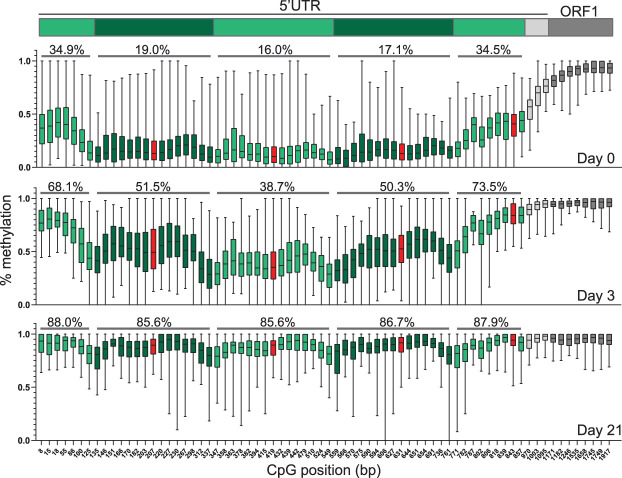
Methylation of individual CpG dinucleotides across the L1 T_F_ promoter. Box-and-whisker plots display the median percent methylation determined by ONT sequencing for individual CpG dinucleotides across the L1 T_F_ promoter for at least 20 individual L1 loci containing five monomer units, at d0 (*top*), d3 (*middle*), and d21 (*bottom*) of differentiation. The CpG positions along the *x*-axis are derived from a representative five monomer L1 T_F_ sequence used in our analysis (Supplemental Table S1). The *central* line represents median percent CpG methylation; box indicates interquartile range. Whiskers represent the *top* and *bottom* quartiles. Alternating green shading indicates CpGs belonging to each monomer unit, corresponding to the schematic of the L1_TF_ 5′ UTR, *above*. The CpG dinucleotide partially encompassed by the core YY1 binding site is shown in red. *Above* each monomer unit for each box plot is shown the average percent methylation among all ≥20 L1 loci across all CpGs present within the monomer unit.

To elucidate the pattern of DNA methylation within the L1 T_FI_ 5′ UTR at single nucleotide resolution, we determined the median percent methylation at each CpG dinucleotide, as well as the average percent methylation for all CpG dinucleotides (%mCpG) within each monomer unit, among at least 20 individual L1 T_FI_ loci containing 2, 3, 4, or 5 monomers, (Fig. 5; Supplemental Fig. 9A–C). This analysis allowed us to quantify the “smile” pattern described above. For example, at day 3 among L1 loci containing five monomer units, the average %mCpG per monomer from the genome-proximal monomer inwards was 68.1%, 51.5%, 38.7%, 50.3%, and 73.5% (Fig. 5, middle panel); a similar pattern was observed for L1 loci containing 2, 3, and 4 monomers (Supplemental Fig. S9). Although methylation was highly variable at most CpG positions, the CpGs flanking the monomer-monomer borders were consistently hypomethylated relative to the CpGs internal to the monomer units. This trend was consistent across L1 T_FI_ elements regardless of monomer count (Fig. 5; Supplemental Fig. S9). We also observed that methylation levels peak at the CpGs flanking the YY1 binding site within each monomer unit, with a slight dip in methylation around the core YY1 binding site. In sum, our ONT methylation analysis of full-length L1s provides unprecedented resolution of interior mouse promoter methylation dynamics.

### DNA methylation during differentiation impacts mouse L1 TSS distribution

**Figure 6.**
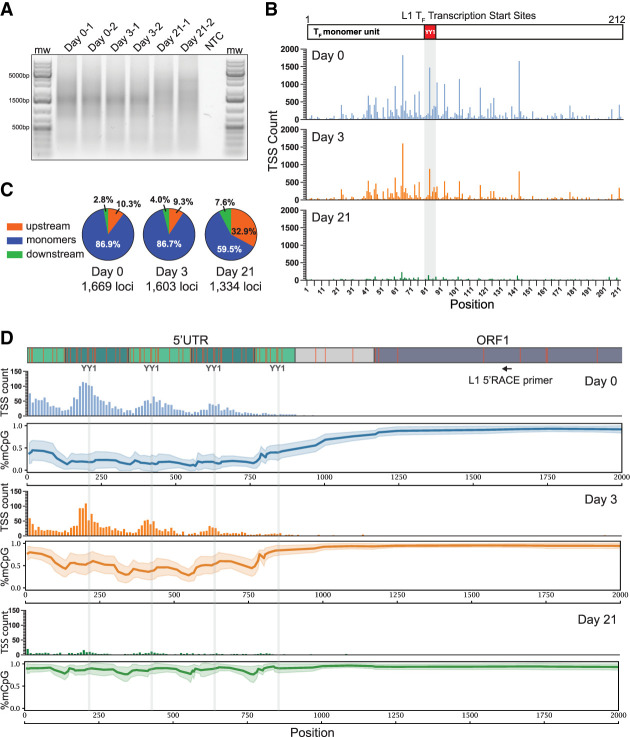
DNA methylation during differentiation impacts mouse L1 TSS distribution. *(A)* Gel image of 5′ RACE products. Total RNA was extracted from two independent replicate cultures at d0 (pluripotent mESCs), d3 of differentiation, and d21 of differentiation and subjected to 5′ RACE NTC; no template control. mw; molecular weight marker. *(B)* Position of TSSs within the L1 T_F_ monomer sequence. The TSS count is shown on the *y*-axis. The position within the 212 bp L1 T_F_ monomer consensus sequence is displayed on the *x*-axis, and a schematic of the L1 T_F_ monomer consensus with the YY1 binding site highlighted in red is shown at the *top*. Blue, d0; orange, d3; green, d21. (*C*) Percentage of TSSs upstream of L1 T_F_ (orange), within L1 T_F_ monomers (blue) and downstream of monomeric region (green) for d0, d3, and d21. The number of loci represented with upstream TSSs at each time point is indicated. (*D*) *Above*, schematic of the first 2000 bp of an L1 T_F_ element containing five monomer units. Alternating green shading represents monomer units. Light gray shading represents the nonmonomeric region of the L1 T_F_ promoter. Dark gray represents ORF1. Orange lines show the position of CpG dinucleotides. The positions of the YY1 binding sites are labeled and represented as vertical light gray lines extending down the figure panel. The position of the L1-specific 5′ RACE primer is indicated. *Below*, TSS counts and mean CpG methylation are shown for time points d0 (blue), d3 (orange), and d21 (green). The histograms in the *upper* plots show TSS count for 137 L1 T_F_ elements with 5 monomer units, with each bar representing a 10 bp bin. The *lower* plots display composite DNA methylation profiles with mean (thick line) and standard deviation (shaded region) indicated.

The fluctuating L1 promoter methylation patterns during mESC differentiation led us to investigate the influence of DNA methylation on L1 T_FI_ TSS usage. We performed 5′ rapid amplification of cDNA ends (5′ RACE) on total RNA isolated from d0, d3, and d21 differentiated mESCs, followed by PCR with an L1 T_FI_ ORF1-specific primer in an approach similar to that used by Deininger et al. to study human L1 expression (Fig. 6A; Deininger et al. 2017). Purified 5′ RACE products were sequenced on a PacBio SMRT flow cell. Reads were filtered to retain those aligned to only one reference genome L1 T_FI_ element, leveraging internal L1 sequence polymorphisms to identify unique TSSs supported by at least one read. At all three time points, TSSs within L1 T_FI_ monomers clustered around the YY1 binding site as previously reported, consistent with our sequence analyses of donor/daughter L1 pairs (Figs. 1C, 6B; DeBerardinis and Kazazian 1999). The most prominent peak at all three time points was at position 65 of the T_F_ monomer consensus sequence, 15 bp upstream of the YY1 binding site (positions 80–87) (Fig. 6B). This finding recalls the situation in the human L1 5′ UTR, in which YY1 directs transcription initiation upstream of the YY1 binding site, near the +1 site of the L1 5′ UTR (Athanikar et al. 2004). Plotting the putative initiator dinucleotide at the −1/+1 position indicated preference for Py/Pu at all three time points (Supplemental Fig. S10A). While in d3 EBs L1 T_FI_ transcripts remained abundant, the total number of TSSs corresponding to L1 T_FI_ elements diminished at d21 (Fig. 6A,B), despite the similar sequencing depth applied to each sample. This potential reduction in L1 T_FI_ transcription at d21 would reconcile with the observed global increase in L1 5′ UTR methylation at this time point (Figs. 4, 5). At all three time points, we identified 5′ RACE products that initiated in genomic sequence upstream of an L1 T_FI_ element; analyzed separately these reads also displayed a preference for a Py/Pu dinucleotide at the −1/+1 position (Supplemental Fig. S10B). Notably, the proportion of upstream initiating transcripts increased substantially in fully differentiated cells at day 21, and the number of loci with reads corresponding to upstream TSSs was 1669 at day 0, 1603 at day 3, 1334 at day 21 (Fig. 6C). The distance between upstream TSSs and their L1s ranged from 1 bp to 420 kb, and the median distance was 163 bp at d0, 115 bp at d3 and 1039 bp at d21. Distances greater than the PacBio read length (mean 1341 bp) likely reflect splicing of the mRNA molecule as previously observed for human L1s (Sanchez-Luque et al. 2019).

We selected two L1 loci with upstream TSSs to examine in greater detail. We analyzed 5′ RACE reads uniquely mapping to Donor 7 (Chr 6: 95,658,065–95,663,747) and observed upstream TSSs at all three time points, with most TSSs located within a SINE B2 element (Supplemental Fig. S11A). Upstream TSSs comprise 30% (20/70) of the reads mapping to this locus at d0, 17% (3/18) at d3, and 63% (5/8) at d21, exemplifying the general shift to upstream TSSs during differentiation. We also examined 5′RACE reads uniquely mapping to a full-length L1 T_F_ element with intact open reading frames (Chr 6: 22,125,162–22,131,805) situated in sense orientation within an intron of the gene *Cped1* (Supplemental Fig. S11B). For this locus, we observed 71% (34/48) upstream TSSs at d0 and 68% (17/25) at d3. At d21, 97% (37/38) of 5′RACE reads for this locus supported upstream TSS usage, with only a single read initiating within the L1 5′UTR. At d0 and d3, most upstream TSSs were located within microsatellite repeats directly adjacent to the L1 element, whereas at d21 most TSSs were located further upstream. Two d21 upstream initiating reads contained exonic *Cped1* sequence and showed evidence of splicing (Supplemental Fig. S11B). Together, these examples suggest that the potential of individual L1 copies to become expressed and retrotranspose is influenced by both their DNA methylation status and their unique genomic environment.

We next generated TSS profiles for the 5′ UTRs of L1 T_FI_ elements containing 2, 3, 4, and 5 monomer units (Fig. 6D; Supplemental Fig. S12A,B). As observed for L1 T_FI_ monomer sequences in general (Fig. 6B), the TSS distribution peaked in the vicinity of the YY1 binding site within each monomer. Notably, we did not observe a TSS peak in the “short” monomer proximal to the nonmonomeric region of the 5′ UTR, despite the presence of a YY1 binding site in this region (Fig. 6D; Supplemental Fig. S12), consistent with recent analysis of L1 T_FI_ promoter activity (Kong et al. 2022). The TSS peaks along the L1 T_FI_ 5′ UTR corresponded to the periodic spikes in DNA methylation, a trend most clearly visible at the day 3 time point (Figs. 4G, 6D; Supplemental Fig. S12). Together, our results detail the dynamics of TSS distribution during establishment of CpG methylation across the mouse L1 T_FI_ promoter.

## Discussion

In this study, we evaluate the mutational capacity of new heritable L1 copies, in terms of their inherent retrotransposition potential and their epigenetic status during development and in adult tissues. We find that loss of 5′ monomers by daughter elements relative to their donors consistently diminishes daughter element retrotransposition efficiency. While for individual examples we cannot determine whether this promoter shortening arose because of 5′ truncation of the L1 cDNA during retrotransposition (Ostertag and Kazazian 2001; Symer et al. 2002; Zingler et al. 2005), or because of the use of a TSS internal to the donor L1 promoter, analysis of putative transcription initiator dinucleotides supports the latter general hypothesis (Fig. 1B). Indeed, L1 T_FI_ TSS distribution analyzed via 5′ RACE suggests that L1 transcription tends to initiate in the vicinity of the YY1 binding site within each monomer, similar to the clustering observed for the first nucleotide of L1 insertions analyzed here and elsewhere (Fig. 1C; DeBerardinis and Kazazian 1999).

Plotting DNA methylation levels determined by ONT sequencing across the L1 5′ UTR revealed a “smile” pattern, wherein the inner monomers tend to be less methylated than the genome-proximal and L1-proximal monomers. This pattern was observed for individual L1 loci (Supplemental Fig. S7) and for the composite methylation profiles of L1s genome-wide, and was most prominent in d3 EBs actively undergoing de novo DNA methylation (Figs. 4G, 6D, 5; Supplemental Figs. S5, S6, S9, S11). Why methylation of the L1 T_F_ promoter apparently proceeds in this “outside-in” fashion is an intriguing topic for future investigations. Our nucleotide resolution analysis of CpG methylation across mouse L1 promoters showed a local peak in CpG methylation in the vicinity of each YY1 binding site (Fig. 5; Supplemental Fig. S9), consistent with a potential role for YY1 in directing the de novo DNA methylation machinery to mouse L1 promoters. Notably, L1 T_F_ TSSs identified by analysis of 5′ RACE products also cluster around the YY1 binding site (Fig. 6B,D; Supplemental Fig. S11), as do the putative TSSs of full-length L1 T_F_ insertions (Fig. 1C; DeBerardinis and Kazazian 1999). Thus, as we speculated previously for human L1s (Sanchez-Luque et al. 2019), YY1 or its binding site(s) within the L1 promoter may be required for both L1 transcription initiation, and, paradoxically, the epigenetic repression of L1 in somatic cells.

Our analysis of L1 TSS distributions revealed that in d21 differentiated cells, the proportion of L1 T_F_ TSSs mapping upstream of the L1 5′ UTR increased compared to d0 and d3, even as the absolute TSS count was diminished, consistent with silencing by DNA methylation (Fig. 6B,C). We speculate that L1 loci residing downstream of promoters that retain activity in differentiated cells are more likely to produce somatic L1 insertions in cells in which the native L1 5′ UTR promoter is heavily methylated. Together with L1 loci that evade 5′ UTR DNA methylation during differentiation (Supplemental Fig. S5), these elements may comprise a cohort of “escapee” loci capable of expression and retrotransposition to generate somatic mosaicism in differentiated cells. Indeed, in our previous studies we encountered somatic L1 insertions that likely arose because of both scenarios (Salvador-Palomeque et al. 2019; Sanchez-Luque et al. 2019; Billon et al. 2022).

Our locus-specific bisulfite sequencing interrogation of donor and daughter L1 methylation in multiple generations of adult tissues, and in differentiating mESCs in vitro, revealed that developmentally active donor L1s and the resultant daughter insertions are generally remethylated in concert with L1 elements genome-wide. De novo daughter L1s were methylated even in the somatic and germ tissues of the mosaic animals in which they arose (Fig. 2), likely because of abundant expression of de novo DNA methyltransferases in pluripotent cells and early postimplantation embryos (Okano et al. 1998; Chen et al. 2003). This result broadly agrees with a previous study analyzing the methylation status of transgene-derived engineered L1 insertions in cultured cells and transgenic animals (Kannan et al. 2017). It should be noted, however, that Kannan et al. queried the methylation status of heterologous promoters or GFP reporter cassette sequences internal to engineered insertions, rather than the mouse L1 5′ UTR. Our ONT analysis across full-length endogenous L1s during differentiation (Fig. 4) showed that the L1 body remains methylated even in pluripotent cells, with the 5′ UTR undergoing dynamic methylation changes likely to affect the expression of full-length L1 transcripts and therefore de novo retrotransposition events. In addition, the “smile” pattern in methylation across the mouse L1 5′ UTR could also lead to an overestimation of mouse L1 promoter methylation, and may impede approaches that analyze only the 5′-most monomers from identifying VM-L1s. Indeed, polyL1Tf_4 is less methylated (<80%) in liver compared to other tissues (>80%) within the same animal, and this methylation pattern is re-established in the F1 but not F2 animal (Fig. 2G). Therefore, it is possible that polyL1Tf_4 is a tissue-specific VM-L1 (Tubio et al. 2014; Schauer et al. 2018; Sanchez-Luque et al. 2019).

In sum, we conclude that the majority of the insertions analyzed here likely arose as a consequence of opportunity provided by genome-wide epigenetic reprogramming during embryonic development (Hajkova et al. 2002; Seki et al. 2005; Tachibana et al. 2007; Abe et al. 2011; Saitou et al. 2012; Seisenberger et al. 2012; Cantone and Fisher 2013). Some exceptional L1s do however escape methylation in a proportion of fully differentiated cells (Supplemental Fig. S5) or, as for Donor 7, appear methylated in somatic tissues but unmethylated in a large fraction of adult germ cells (Fig. 2E,H; Supplemental Fig. S2A,C). Indeed, a recent study examining the somatic L1 epigenetic and retrotransposition landscape in human colorectal epithelial cell clones concluded that L1 methylation escape occurs postgastrulation during the early stages of organogenesis (Nam et al. 2023). Future studies are likely to reveal additional tissue and developmental stage-specific mouse L1 “escapee” loci, with the capacity to evade genome-wide methylation by virtue of their sequence content or surrounding genomic environment.

## Methods

### Mice

All mouse work was performed in compliance with the guidelines set forth by the University of Queensland Animal Ethics Committee. Tissues from animals generated for Richardson et al. (2017) were used for this study.

### Cell culture

HeLa-JVM cells were maintained at 37°C and 5% CO_2_ in Dulbecco's Modified Eagle's Media (DMEM) (Thermo Fisher Scientific) supplemented with 10% heat-inactivated fetal bovine serum (FBS) (Thermo Fisher Scientific), 1% L-glutamine (Thermo Fisher Scientific) and 1% penicillin-streptomycin (Thermo Fisher Scientific). The cells were passaged every 3–4 d after they achieved confluency of 80%–90% using Trypsin 0.25% EDTA (Thermo Fisher Scientific).

E14Tg2a mESCs (ATCC CRL-1821) were cultured on gelatinized tissue culture plates and maintained at 37°C and 5% CO_2_. Cells were maintained in serum + LIF, 2i + serum, and 2i + LIF conditions and were passaged at 70%–80% confluence every 2–3 d using Trypsin 0.25% EDTA (Thermo Fisher Scientific), with media changed every day. The details of mESC culture conditions and media composition are provided in the Supplemental Methods. E14Tg2a mESCs were differentiated into EBs using the hanging drop method as previously described (Behringer et al. 2016). Details of the mESC differentiation protocol can be found in the Supplemental Materials.

### Identification of L1 donor/daughter pairs

We previously identified 11 de novo endogenous L1 T_F_ insertions among 85 C57BL6/J mice belonging to multigeneration pedigrees, as well as six unfixed polymorphic L1 T_F_ insertions absent from the C57BL6/J reference genome and differentially present/absent among these pedigrees (Richardson et al. 2017). We traced the majority of heritable insertions to pluripotent embryonic cells, evidenced by shared somatic/germline mosaicism of the founder mouse, and early primordial germ cells (PGCs), evidenced by germline-restricted mosaicism across both testes of male founder mice. Analysis of unique L1 3′ transduced sequences (Holmes et al. 1994; Moran et al. 1999; Goodier et al. 2000; Pickeral et al. 2000; Xing et al. 2006) allowed us to identify the source (donor) L1 loci responsible for three offspring (daughter) de novo insertions (one early embryonic, two early PGC) and two unfixed polymorphic daughter insertions.

### DNA extraction

Genomic DNA from mouse tissue was extracted as previously described (Richardson et al. 2017).

Genomic DNA from cultured cells was extracted using DNeasy Blood & Tissue kit (Qiagen) according to the manufacturer's protocol. The DNA concentration was determined by Qubit 3.0 Fluorometer (Invitrogen) using the Qubit dsDNA HS Assay Kit (Invitrogen) following the manufacturer's instructions.

High molecular weight (HMW) genomic DNA from cultured cells was extracted using the Nanobind CBB Big DNA Kit (Circulomics) following the manufacturer's instructions.

### Generation of mouse L1 reporter constructs

Mouse L1 reporter constructs were generated via PCR amplification of donor/daughter mouse L1s from genomic DNA, followed by capillary sequencing of multiple clones to identify PCR errors. Constructs containing the full, correct L1 sequence were built from individual PCR clones as described previously (Sanchez-Luque et al. 2019). A modified version of the previously described pTN201 construct (Naas et al. 1998) in which the L1 3′ UTR polypurine tract is located downstream, rather than upstream, of the NEO indicator cassette was used as a backbone to generate L1 reporter constructs (Richardson et al. 2022). The molecular details of the cloning strategy are presented in the Supplemental Methods.

### Immunostaining

Immunostaining was performed on day 7 (24 h after plating of EBs) and day 21 of mESC differentiation. EBs were plated on gelatinized coverslips in twelve-well plates. Immunostaining was performed using primary antibodies against β-tubulin (Rabbit IgG; Sigma-Aldrich, #T2200), Α-fetoprotein (AFP), (Goat IgG; R&D Systems AF5369), and smooth muscle actin (Mouse IgG; Thermo Fisher Scientific 14976080). Secondary antibodies used were Alexa Fluor 647 Donkey Anti-Rabbit IgG (Jackson ImmunoResearch 711-606-152); Cy3 Donkey Anti-Goat IgG (Jackson ImmunoResearch 715-165-150), and Alexa Fluor 488 Donkey Anti-Mouse IgG (Jackson ImmunoResearch 715-546-150). Details of the immunostaining and imaging procedures are supplied in the Supplemental Methods.

### Retrotransposition assay

Retrotransposition assays in HeLa-JVM cells were performed as previously described (Kopera et al. 2016) with some minor modifications. HeLa-JVM cells were plated at the appropriate density for each construct and transfected the following day with 1 ug of plasmid DNA per well using FuGene-HD (Promega). Transfection efficiency was determined in parallel using L1 reporter construct and pCEP4-eGFP co-transfection. Geneticin/G418 (400 μg/mL) (Thermo Fisher Scientific) selection was started 3 d post-transfection and performed for 12 d, after which the cells were fixed and stained with crystal violet. Colonies were counted and retrotransposition efficiency determined by normalizing colony counts to transfection efficiency for each construct. The full details of the HeLa-JVM retrotransposition assay protocol are provided in the Supplemental Methods.

L1 retrotransposition assays in E14Tg2a mESCs (ATCC CRL-1821) were performed as previously described (MacLennan et al. 2017) in antibiotic-free 2i + serum conditions using 1 μg of plasmid DNA per well of a six-well dish and Lipofectamine 2000 (Invitrogen) according to the manufacturer's instructions. 24 h after transfection, the mESCs were passaged into 10 cm dishes. G418 (Invitrogen) selection (200 μg/mL) was started 24 h after passaging and continued for 12 d. Drug-resistant colonies were fixed, stained and counted as described for HeLa-JVM cells. The full details of the mESC retrotransposition assay protocol are provided in the Supplemental Methods.

### Locus-specific bisulfite sequencing

Bisulfite conversion was performed using the EZ DNA Methylation-Lightning Kit (Zymo Research), following the manufacturer's instructions. Primers used for target amplification are listed in Supplemental Table S1. PCRs were performed using MyTaq HS DNA Polymerase (Bioline). PCR products were gel purified using the MinElute Gel Extraction Kit (Qiagen) according to the manufacturer's instructions. Illumina libraries were constructed using the NEBNext Ultra II DNA Library Prep Kit (New England Biolabs) following the manufacturer's instructions. Barcoded libraries were pooled equimolar and sequenced on an Illumina MiSeq platform using a MiSeq Reagent Kit v3 (600-cycle). Analysis was performed as previously described (Schauer et al. 2018). Unconverted amplicon sequences are listed in Supplemental Table S1. Per sample, up to 1000 reads per violin plot were randomly extracted from the bisulfite sequencing files using the ExSeq tool (https://github.com/MischaLundberg/extract_sequences). Selected reads were analyzed using QUMA (QUantification tool for Methylation Analysis) (Kumaki et al. 2008). Full details of the locus-specific bisulfite sequencing analysis can be found in the Supplemental Methods.

### Nanopore sequencing

Purity of HMW DNA was determined using a NanoDrop One Spectrophotometer (Thermo Fisher Scientific) according to the manufacturer's instructions. The DNA concentration was determined by Qubit 3.0 Fluorometer (Invitrogen) using the Qubit dsDNA HS Assay Kit (Invitrogen) and on a TapeStation System (Agilent Technologies). ONT sequencing libraries were created using 1D Ligation (SQK-LSK109), sheared to create an average fragment size of ∼10 kb and sequenced at the Kinghorn Centre for Clinical Genomics at the Garvan Institute of Medical Research (Darlinghurst, NSW, Australia) on an ONT PromethION platform.

Bases were called using Guppy version 3.2.10 (Oxford Nanopore Technologies) and aligned to the reference genome build mm10 using minimap2 version 2.17 (Li 2018) and SAMtools version 1.3 (Li et al. 2009). Reads were indexed and per-CpG methylation calls generated using nanopolish version 0.13.2 (Simpson et al. 2017). Methylation likelihood data were sorted by position and indexed using tabix version 1.10.2 (Li 2011).

Reference L1 locations were derived from the RepeatMasker (https://www.repeatmasker.org) .OUT track files available for mm10 from the UCSC Genome Browser (Kent 2002). As full-length mouse L1s are often broken into multiple adjacent annotations when present on the - strand of the genome, we merged adjacent similarly oriented L1s before analysis. L1s were considered if annotated as >6000 bp in length. Monomers were counted using a Python script which considers the best alignment between a library of known monomer sequences and a target transposable element using exonerate (Slater and Birney 2005), records and masks the monomer alignment, and repeats the process until no further monomer alignments are present. Methylation results were annotated per-subfamily and per-monomer count and plotted using these categories. Methylation statistics for reference L1s were generated using the “segmeth” function in methylartist (https://github.com/adamewing/methylartist) and plotted using the “segplot” function. Reads mapping completely within L1s were excluded from the reference L1 methylation analysis via the “‐‐exclude_ambiguous” option in methylartist segmeth to negate the contribution of ambiguous mappings. Plots categorized by monomer count (Fig. 4D) were limited to reads spanning the entire segment with the addition of the “‐‐spanning_only” argument to segmeth. Methylation plots for individual L1s and differentially methylated regions (DMRs) (Fig. 5) were created using the methylartist “locus” function. Composite per-element methylation plots (Fig. 4F,G; Supplemental Figs. S5, S7) were created using the methylartist “composite” function, with individual CpG statistics obtained via the –output_table and –meanplot_cutoff 20 parameters.

### 5′ RACE

Total RNA was extracted from E14Tg2a mESCs differentiated to embryoid bodies as described above, at day 0, day 3, and day 21 of the differentiation protocol using the RNeasy Mini kit (Qiagen, #74104). 5′ RACE was performed using the 5′RACE module of the SMARTer 5′/3′ RACE Kit (Takara Bio, #634858) according to the manufacturer's protocol. 1 µg total RNA was used as input for each reaction, and two reactions from biologically independent samples were performed per time point. PCR amplification of 5′ RACE cDNAs was performed using the L1 gene specific primer 5′-catctcttgtattctgttgctgatgctcaa-3′ and the 5′ RACE universal primer provided in the SMARTer 5′/3′ RACE Kit (Takara Bio). 25 PCR cycles were performed as follows: 94°C for 30 sec, 67 °C for 30 sec, 72 °C for 3 min. PCR products were visualized on a 1% agarose gel. Products ranging from 700–5000 bp were excised, and PCR fragments were purified using the MinElute Gel Extraction Kit (Qiagen, # 28604). Iso-Seq template preparation using the Iso-Seq Express kit followed by PacBio SMRT Cell sequencing on a PacBio Sequel II platform was performed by the Australian Genome Research Facility. Six samples (two replicates per time points d0, d3, and d21) were multiplexed on a single SMRT cell, generating 3,699,664 CCS reads in total.

Reads were aligned to the mouse reference genome (mm10) using minimap2 (Li 2018) and parameters (-t 96 -N 1000 -p 0.95 -ax splice:hq -uf) and sorted with SAMtools (Li et al. 2009). Uniquely mapped reads, that is, those that aligned to one genomic position only at their best alignment score and as a primary alignment, were retained if an L1-specific primer and a 5′ RACE universal primer were located at each of their termini. Multimapping reads were discarded to enable unambiguous read assignment to a given monomer-count resolved L1. The majority (>75%) of reads aligned to an L1 also aligned uniquely to the genome. Reads were then assigned to the full-length L1 T_FI_ elements they overlapped, with alignments required to terminate within the L1 body. The start positions of these alignments within the 5′ UTR or upstream of the L1 were then recorded as putative TSSs supported by at least one read. These positions were used to generate TSS distributions relative to monomer coordinates or to L1 T_FI_ 5′ UTRs composed of 2, 3, 4 or 5 monomers. Sequence logos surrounding these TSSs were generated using WebLogo (Crooks et al. 2004).

## Data access

All Sanger sequencing data generated in this study have been submitted to the NCBI GenBank database (https://www.ncbi.nlm.nih.gov/genbank/) under accession numbers OQ856744–OQ856753.

The Oxford Nanopore Technologies and PacBio data generated in this study have been submitted to the NCBI BioProject database (https://www.ncbi.nlm.nih.gov/bioproject/) under accession number PRJNA763783.

New scripts TSSprofile.py and intersect.py used to analyse TSS distribution across L1 T_F_ elements with 2, 3, 4, and 5 monomers have been uploaded as Supplemental Code.

## Supplementary Material

Supplement 1

Supplement 2

Supplement 3

Supplement 4

Supplement 5

Supplement 6

Supplement 7

Supplement 8

Supplement 9

Supplement 10

Supplement 11

Supplement 12

Supplement 13

Supplement 14

Supplement 15
