# Supplementary material for: Locus-resolution analysis of L1 regulation and retrotransposition potential in mouse embryonic development
Source: Genome Res. 2023 Sep;33(9):1465–81. doi: 10.1101/gr.278003.123 (PMC10620060; doi:10.1101/gr.278003.123)
Supplement: Supplement 1 [file Supplemental_Fig_S1.pdf]

Supplemental Figure S1

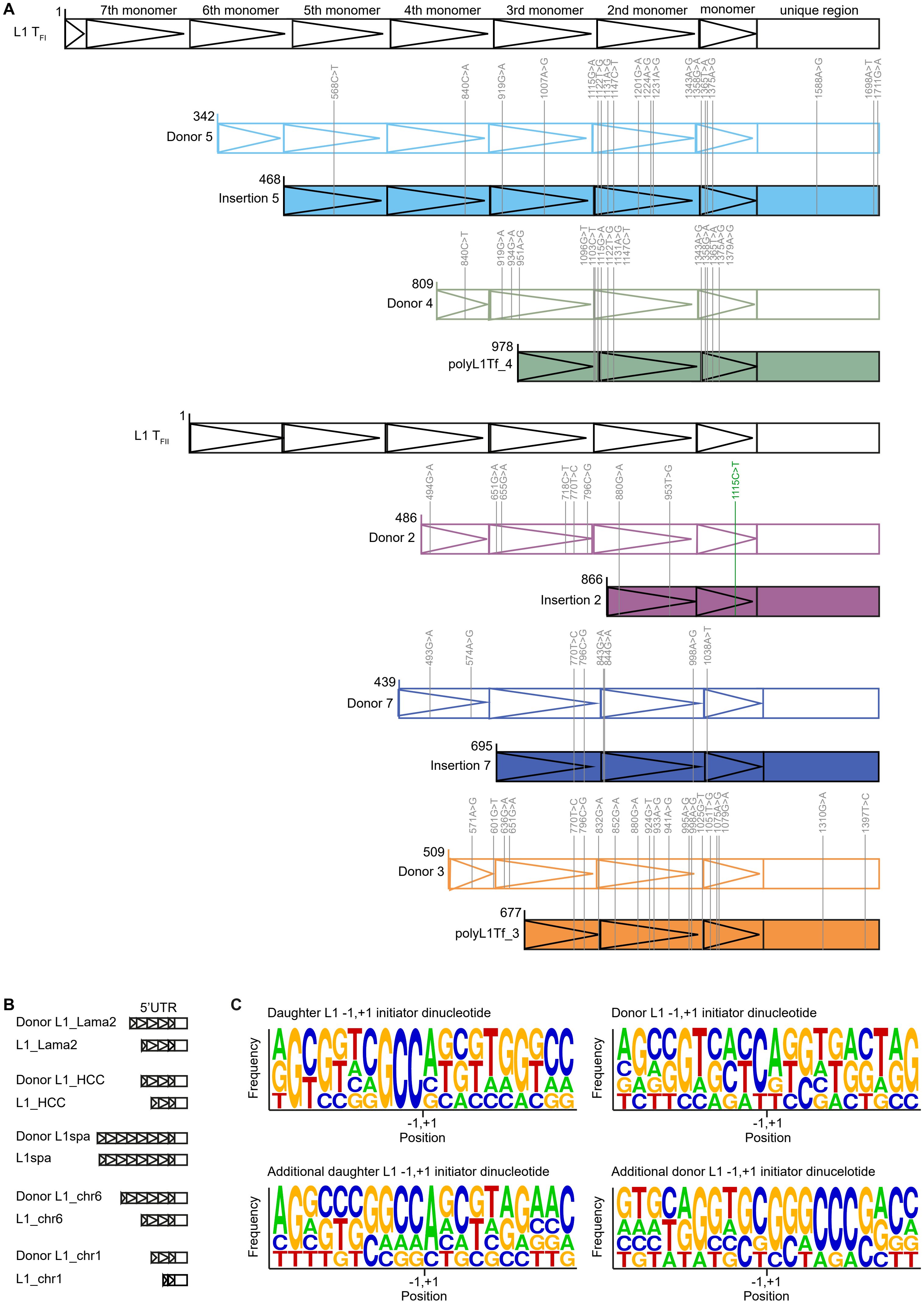

Supplemental Figure S1. L1 donor/daughter promoter sequence comparison.

(*A*) Sequence comparison of L1 T_{FI} and T_{FII} donor/daughter pair promoters. Nucleotide substitutions compared to the consensus sequence are annotated in grey. Nucleotide substitution in green represents mutation within the extended YY1 binding motif. Triangles within 5′ UTR represent monomer units.

(*B*) Cartoons for promoters of 5 additional L1 donor/daughter pairs identified in the literature and our analysis.

(*C*) Sequence logos (Crooks et al. 2004) of putative transcription initiation start sites for donor and daughter L1s. The top two plots represent the five daughter (left) and donor (right) elements identified in Richardson et al. 2017 (Richardson et al. 2017). The bottom two plots represent the five additional daughter (left) and donor (right) elements identified in the literature and our analysis. Sequence represents -1,+1 transcription initiator dinucleotide in the center ±9 nucleotides upstream and downstream. +1 indicates first nucleotide of L1 sequence which corresponds to second nucleotide in transcription initiator dinucleotide.
