# Supplementary material for: Locus-resolution analysis of L1 regulation and retrotransposition potential in mouse embryonic development
Source: Genome Res. 2023 Sep;33(9):1465–81. doi: 10.1101/gr.278003.123 (PMC10620060; doi:10.1101/gr.278003.123)
Supplement: Supplement 2 [file Supplemental_Fig_S2.pdf]

Supplemental Figure S2

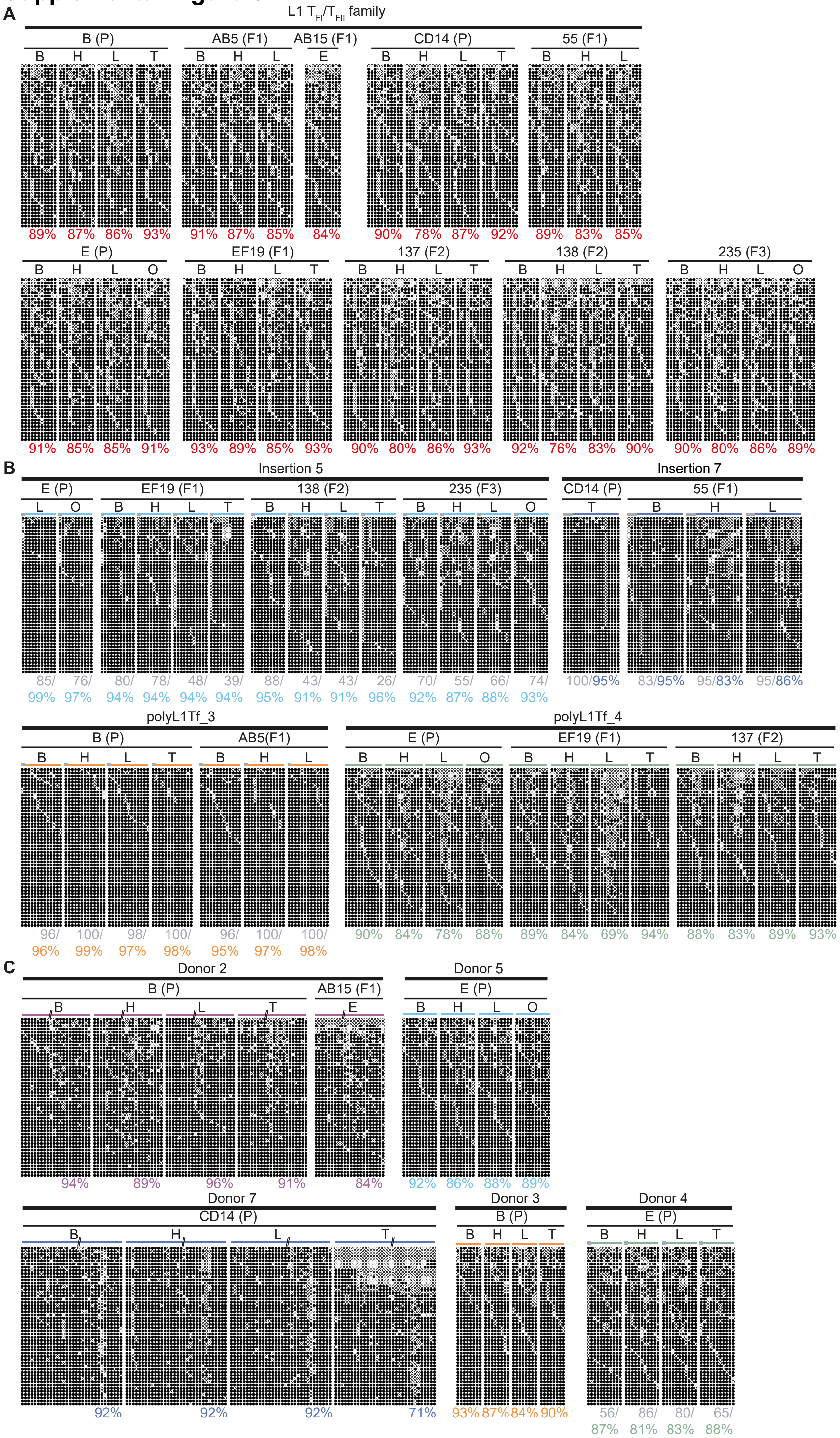

Supplemental Figure S2. Methylation of L1 T_{FI}/ T_{FII} family and L1 donor/daughter pairs in mouse tissues.

(*A*) Genome-wide methylation level of CpG dinucleotides of L1 T_{FI}/ T_{FII} promoter sequence is shown. Displayed are 50 non-identical sequences extracted at random from a much larger pool of available Illumina reads. Each cartoon panel corresponds to an amplicon (black circle, methylated CpG; white circle, unmethylated CpG; ×, mutated CpG). The overall percentage of methylated CpG dinucleotides is indicated below each cartoon.

(*B*) As for (*A*) except for methylation level of CpG dinucleotides of L1 daughter insertions is shown. Colored line above the cartoon represents amplicon (grey, genomic sequence; colored, L1 sequence). Grey letters indicate overall percentage of methylated CpG dinucleotides in genomic sequence. Colored letters indicate methylation of CpG dinucleotides in L1 sequence.

(*C*) As for (*A*) and (*B*) but for donor L1s. The promoters of Donor 2 and Donor 7 were not completely sequenced and missing the center monomer as indicated by black lines in colored line above methylation cartoons.
