# Supplementary material for: Locus-resolution analysis of L1 regulation and retrotransposition potential in mouse embryonic development
Source: Genome Res. 2023 Sep;33(9):1465–81. doi: 10.1101/gr.278003.123 (PMC10620060; doi:10.1101/gr.278003.123)
Supplement: Supplement 3 [file Supplemental_Fig_S3.pdf]

Supplemental Figure S3

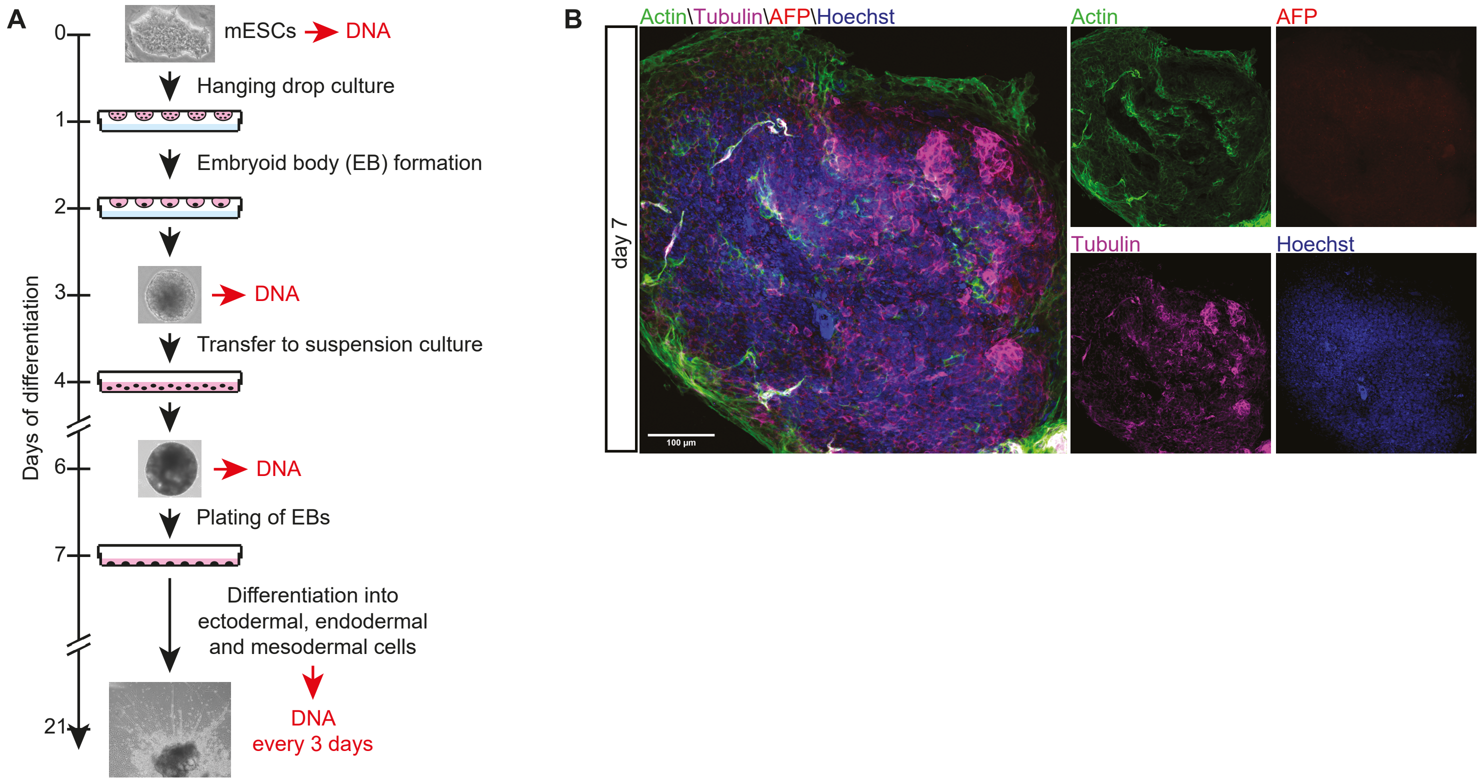

Supplemental Figure S3. Differentiation of E14 mESCs.

(*A*) Schematic of mESC differentiation by hanging drop culture. Differentiation of mESCs was induced by culturing mESCs in hanging drops to induce embryoid body (EB) formation. On day three EBs were transferred to low-attachment tissue culture dishes. After 6 days, EBs were plated and differentiated for 14 days. On day 21, differentiated cells were fixed and stained for markers of ectodermal, endodermal and mesodermal cells. Cells were collected every 3 days during differentiation and DNA for methylation analysis was extracted.

(*B*) Immunofluorescence image of mesodermal (Actin), endodermal (AFP) and ectodermal (Tubulin) lineage markers in differentiated E14 mESCs on d7 (one day after plating EBs). Nuclei were stained with Hoechst (blue). Scale bar, 100 µm.
