# Supplementary material for: Locus-resolution analysis of L1 regulation and retrotransposition potential in mouse embryonic development
Source: Genome Res. 2023 Sep;33(9):1465–81. doi: 10.1101/gr.278003.123 (PMC10620060; doi:10.1101/gr.278003.123)
Supplement: Supplement 4 [file Supplemental_Fig_S4.pdf]

Supplemental Figure S4

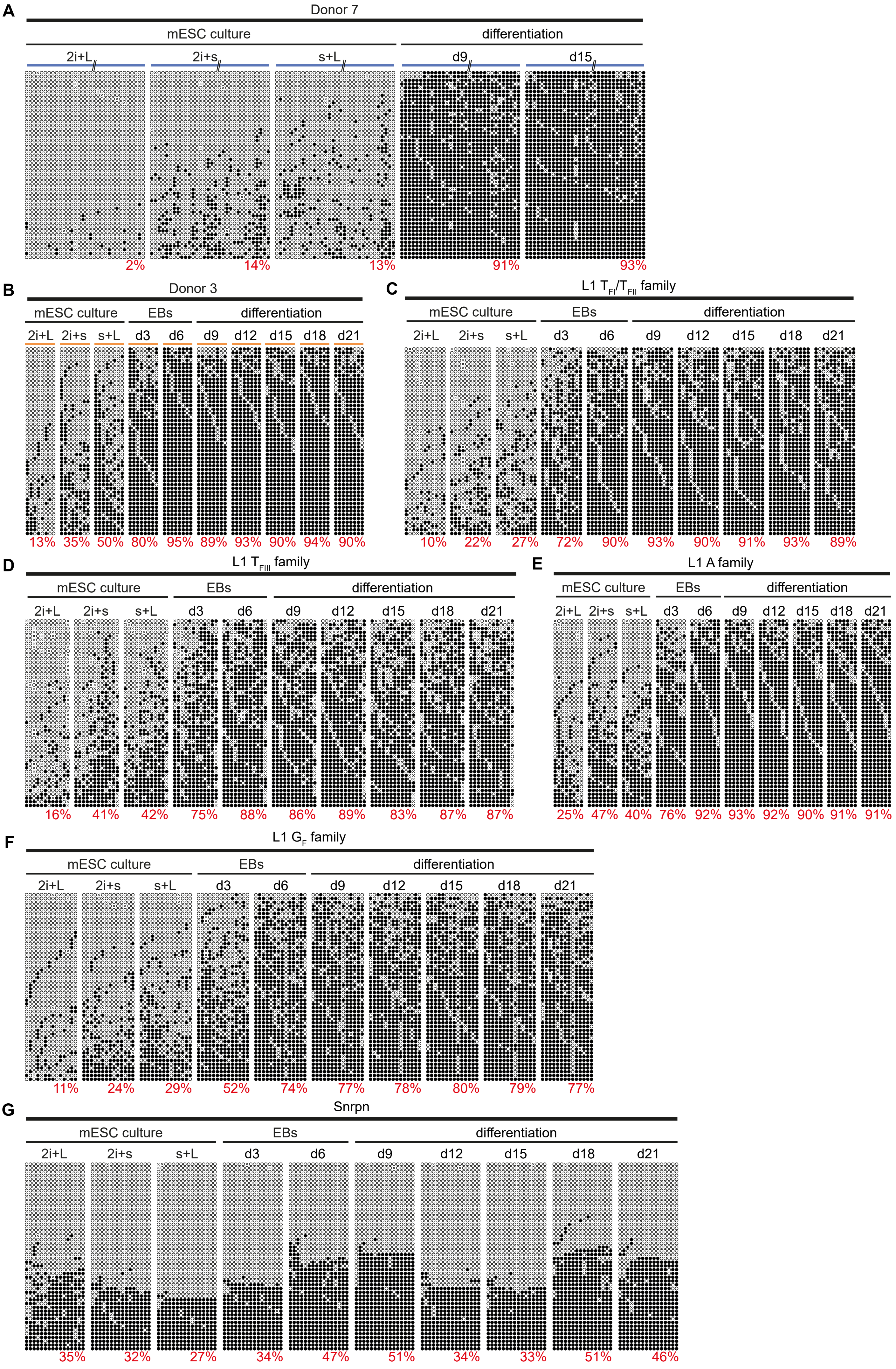

Supplemental Figure S4. Dynamic methylation during mESC differentiation.

(*A*) Methylation of Donor 7 promoter sequence shown in the mESCs cultured in three different conditions (2i+L = 2i+LIF, 2i+s = 2i+serum, s+L = serum+LIF) and on differentiation day 9 (d9) and day 15 (d15). Displayed are 50 non-identical sequences extracted at random from a much larger pool of available Illumina reads. Each cartoon panel corresponds to an amplicon (black circle, methylated CpG; white circle, unmethylated CpG; ×, mutated CpG). Colored line above each cartoon represents amplicon (grey = genomic sequence, colored = L1 sequence). The overall percentage of methylated CpG dinucleotides is indicated below each cartoon. Grey letters indicate methylation of CpG dinucleotides in genomic sequence. Colored letters indicate methylation of CpG dinucleotides in L1 sequence. The promoter of Donor 7 was not completely sequenced as indicated by black lines in colored line above methylation cartoons.

(*B-F*) As per (*A*) but for Donor 3 (*B*), L1 T_{FI}/T_{FII} family (*C*), L1 T_{FIII} family (*D*), L1 A family (*E*), L1 G_F family (*F*). Primers for L1 subfamilies are within the L1 promoter sequence. Shown is methylation in three different mESC culture conditions, during EB culture and during differentiation.

(*G*) As per above but for the imprinted gene *Snrpn*.
