# Supplementary material for: Locus-resolution analysis of L1 regulation and retrotransposition potential in mouse embryonic development
Source: Genome Res. 2023 Sep;33(9):1465–81. doi: 10.1101/gr.278003.123 (PMC10620060; doi:10.1101/gr.278003.123)
Supplement: Supplement 5 [file Supplemental_Fig_S5.pdf]

Supplemental Figure S5

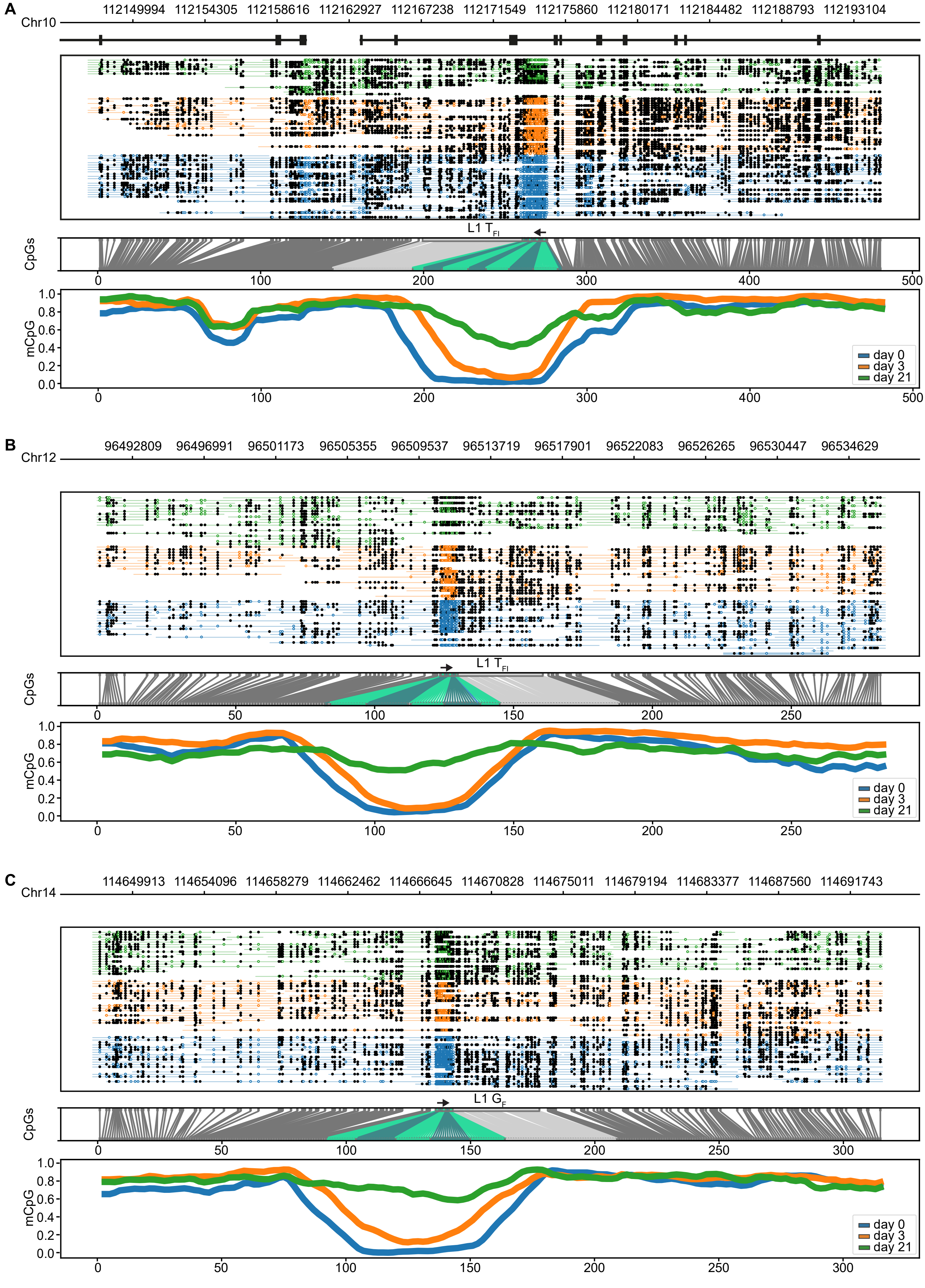

Supplemental Figure S5. ONT methylation profiles for L1 somatic methylation "escapee" loci.

(*A*) Methylation of an L1 T_{FI} element on chromosome 10 and surrounding locus. From *top* to *bottom* this figure shows: i) the genomic position of the L1 in an intron on chromosome 10, including 20 kbp up- and downstream of the L1; ii) a diagram showing methylated (filled black circles) and unmethylated (unfilled colored circles) CpGs and read (colored lines) coverage per sample; iii) a diagram displaying the correspondence between genome space and CpG space, CpGs belonging to full-length L1 are annotated in light and dark green (promoter monomers) and light grey (ORFs and 3′ UTR); iv) the fraction of methylated CpGs for three differentiation time points (d0, d3, d21) in CpG space.

(*B*) As for (*A*) except for an intergenic L1 T_{FI} element on chromosome 12.

(*C*) As for (*A*) except for an intergenic L1 G_F element on chromosome 14.
