# Supplementary material for: Locus-resolution analysis of L1 regulation and retrotransposition potential in mouse embryonic development
Source: Genome Res. 2023 Sep;33(9):1465–81. doi: 10.1101/gr.278003.123 (PMC10620060; doi:10.1101/gr.278003.123)
Supplement: Supplement 6 [file Supplemental_Fig_S6.pdf]

Supplemental Figure S6

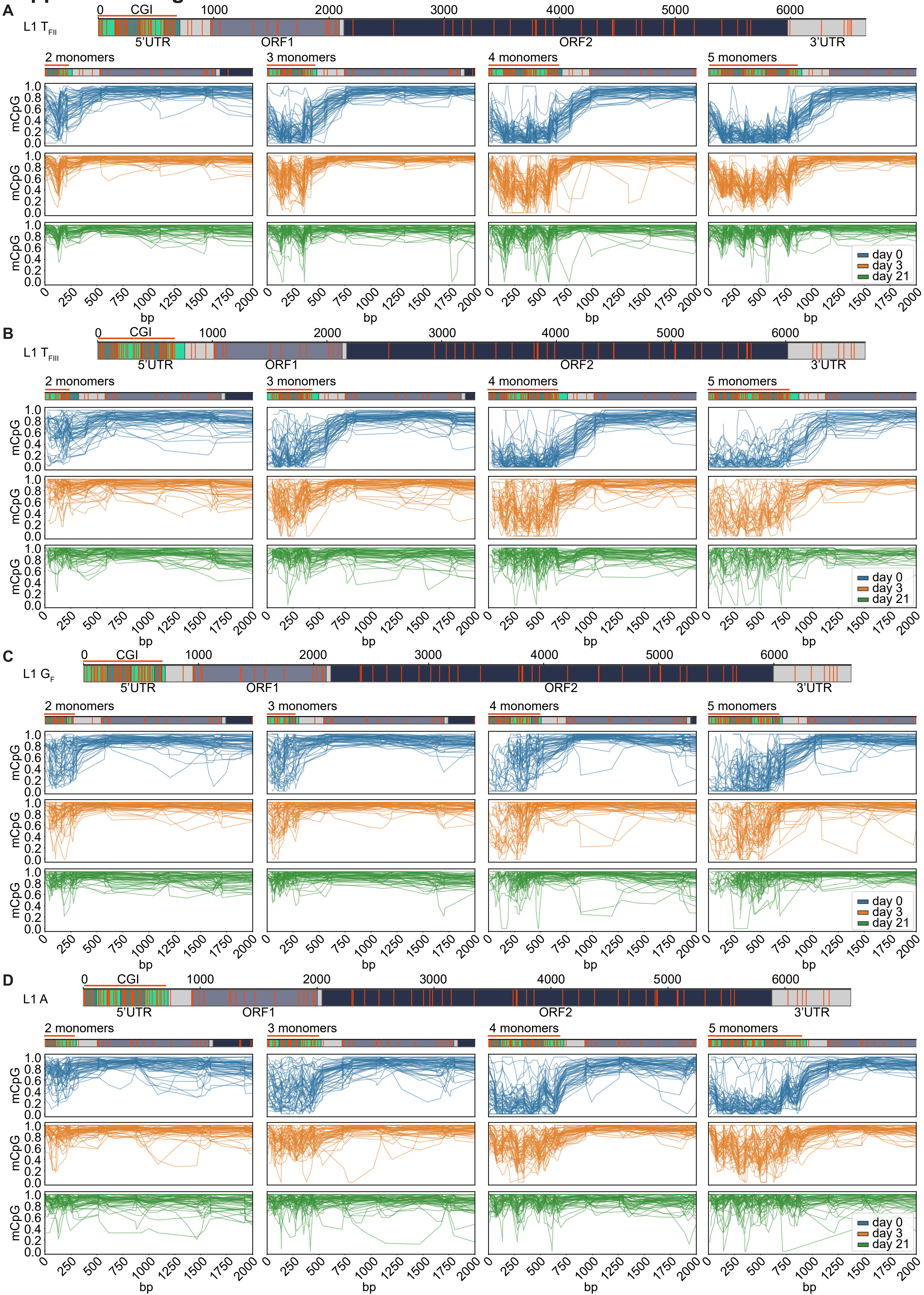

Supplemental Figure S6. Composite methylation profiles of L1 subfamily promoters during differentiation.

(*A*) *Top*: Annotated full-length L1 T_{FII} consensus showing the monomer units in green, unique region in light grey, ORF1 in dark grey, ORF2 in dark green, and 3′ UTR in light grey. CpG dinucleotides throughout the whole element are displayed as orange strokes. The promoter CpG island (CGI) is indicated as an orange line. Number of bp are shown above the element.
Bottom: Data is shown for L1 T_{FII} promoters containing 2, 3, 4 and 5 monomers at three time points of differentiation: d0 (undifferentiated mESCs in serum+LIF), d3 (EBs on day 3 of differentiation) and on d21 (completely differentiated cells). Each graph displays up to 50 methylation profiles. Annotated consensus sequences as per (*top*) are shown at *top* including CpG positions.

(*B*) As for (*A*) except for L1 T_{FIII} promoters.

(*C*) As for (*A*) except for L1 G_F promoters.

(*D*) As for (*A*) except for L1 A promoters.
