# Supplementary material for: Locus-resolution analysis of L1 regulation and retrotransposition potential in mouse embryonic development
Source: Genome Res. 2023 Sep;33(9):1465–81. doi: 10.1101/gr.278003.123 (PMC10620060; doi:10.1101/gr.278003.123)
Supplement: Supplement 7 [file Supplemental_Fig_S7.pdf]

Supplemental Figure S7

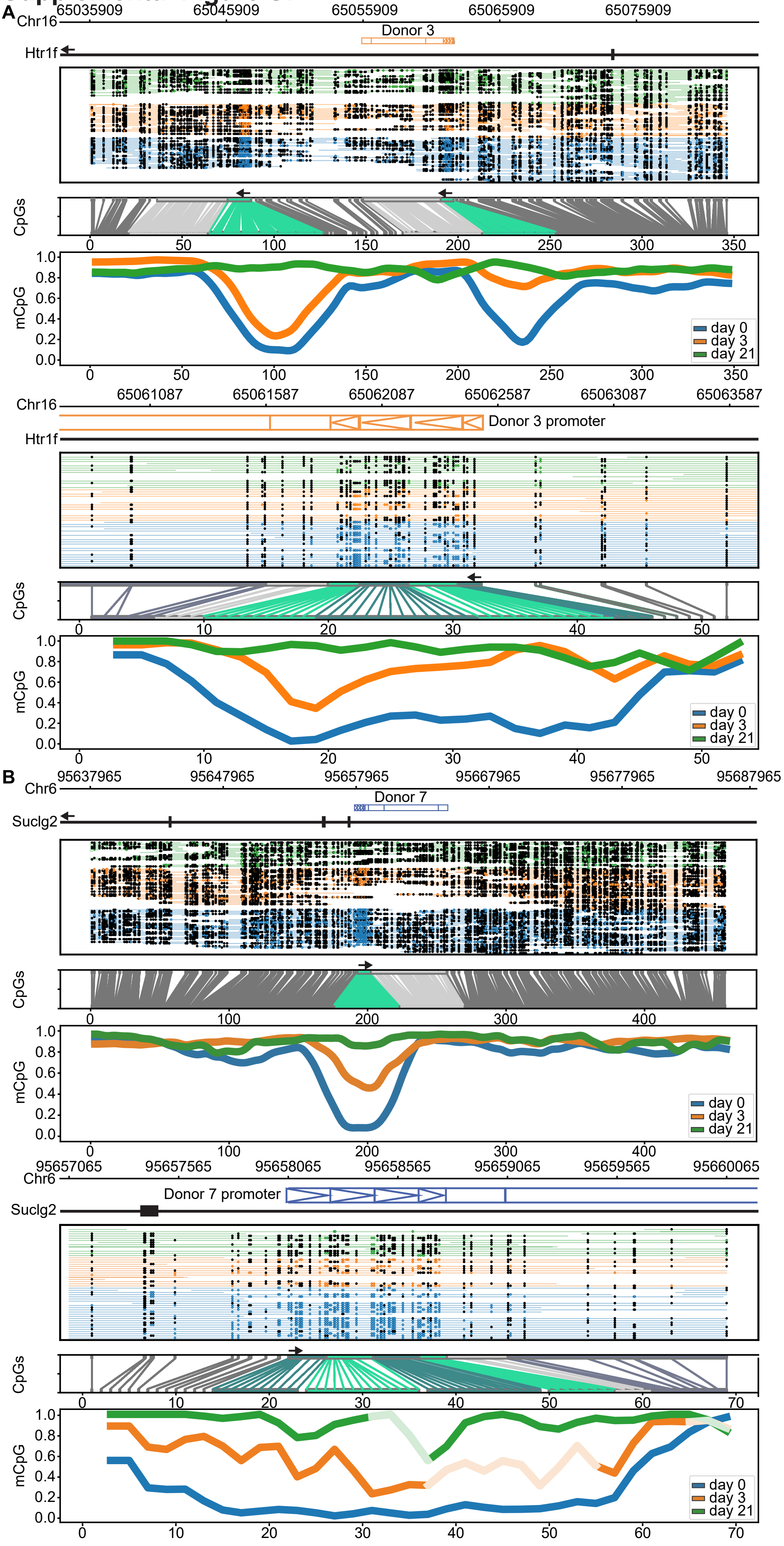

Supplemental Figure S7. Donor 3 and Donor 7 ONT methylation profiles during mESC differentiation.

(*A*) *Top*: Methylation of Donor 3 and surrounding locus. From *top* to *bottom* this figure shows i) the genomic position of Donor 3 in an intron of the *Htr1f* gene on chromosome 16, including 20 kbp up- and downstream of Donor 3, ii) a diagram showing methylated (filled black circles) and unmethylated (unfilled colored circles) CpGs and read (colored lines) coverage per sample, iii) a diagram displaying the correspondence between genome space and CpG space, CpGs belonging to full-length L1 are annotated in light green (promoters) and light grey (ORFs and 3′ UTR), iv) the fraction of methylated CpGs for three differentiation time points (d0, d3, d21) in CpG space. *Bottom*: as for upper panels but for promoter of Donor 3, including 1 kbp up- and downstream of the promoter with less smoothing. Monomers in iii) are annotated in light and dark green, unique region in light grey, the start of ORF1 in dark grey/blue. Graphs are shown via a sliding window plot.

(*B*) As for (*A*) but for Donor 7 and surrounding locus. The Donor 7 promoter (*bottom* panel (*B*)) smoothed plot lines are colored to appear faded for a short lower confidence region (<20 methylated/demethylated calls within a 30 CpG window).
