# Supplementary material for: Locus-resolution analysis of L1 regulation and retrotransposition potential in mouse embryonic development
Source: Genome Res. 2023 Sep;33(9):1465–81. doi: 10.1101/gr.278003.123 (PMC10620060; doi:10.1101/gr.278003.123)
Supplement: Supplement 8 [file Supplemental_Fig_S8.pdf]

Supplemental Figure S8

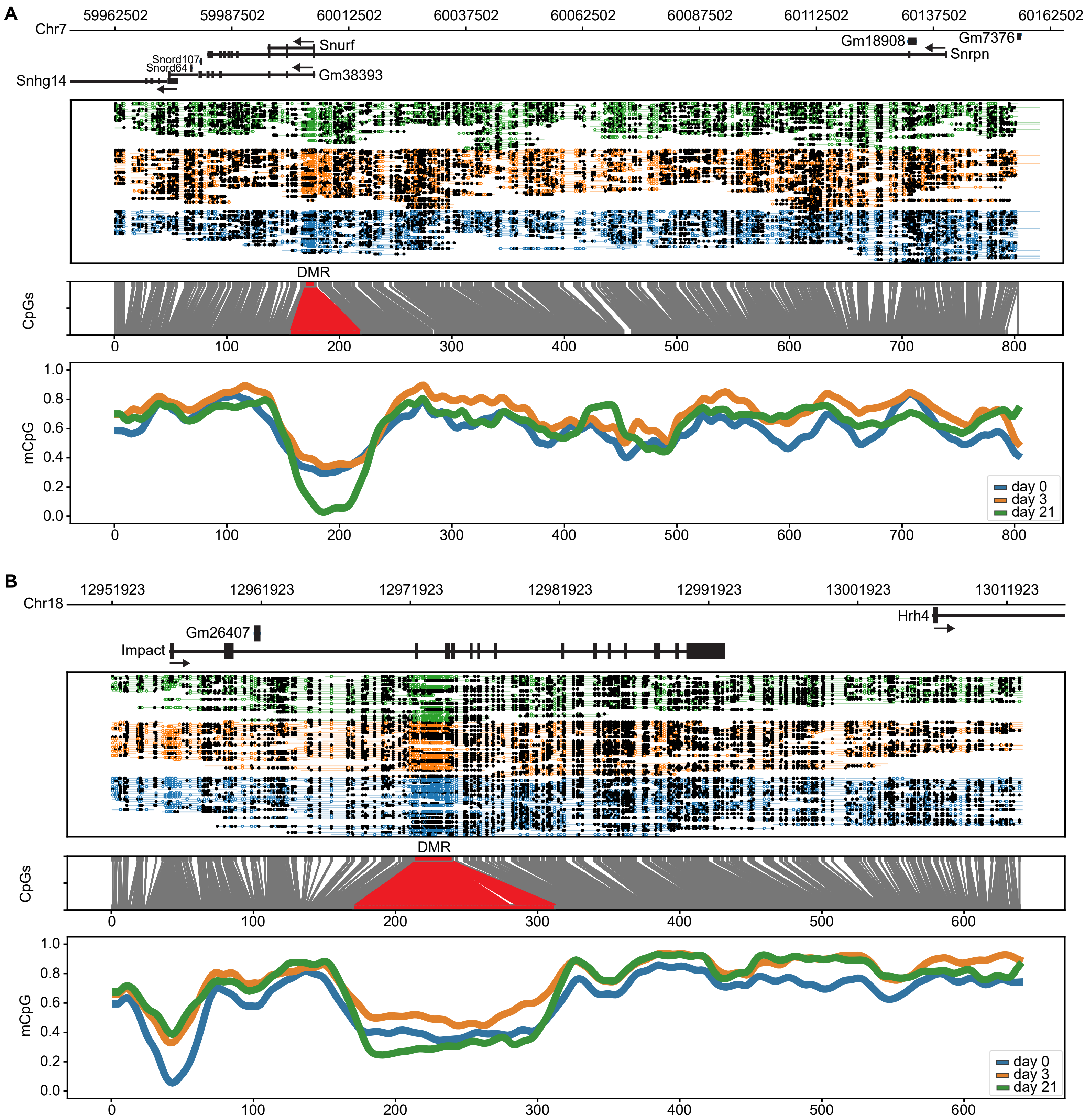

Supplemental Figure S8. ONT methylation profiles of imprinted genes *Snrpn* and *Impact*.

(*A*) Methylation of the *Snrpn* gene and surrounding locus. From *top* to *bottom* this figure shows i) the genomic position of *Snrpn* on chromosome 7, including 20 kbp up- and downstream of *Snrpn*, ii) a diagram showing methylated (filled black circles) and unmethylated (unfilled colored circles) CpGs and read (colored lines) coverage per sample, iii) a diagram displaying the correspondence between genome space and CpG space, CpGs belonging to the *Snrpn* DMR are annotated in red, iv) the fraction of methylated CpGs for three differentiation time points (d0, d3, d21) in CpG space. The low methylation over the *Snrpn* DMR is likely a sampling issue due to low coverage. Because the E14 mESCs used in this experiment are derived from an inbred mouse strain, it is not possible to phase the methylation profiles of individual reads to confirm that only one allele of the maternally imprinted *Snrpn* DMR has been sampled.

(*B*) As for (*A*) except for the imprinted *Impact* gene.
