# Supplementary material for: Locus-resolution analysis of L1 regulation and retrotransposition potential in mouse embryonic development
Source: Genome Res. 2023 Sep;33(9):1465–81. doi: 10.1101/gr.278003.123 (PMC10620060; doi:10.1101/gr.278003.123)
Supplement: Supplement 9 [file Supplemental_Fig_S9.pdf]

Supplemental Figure S9

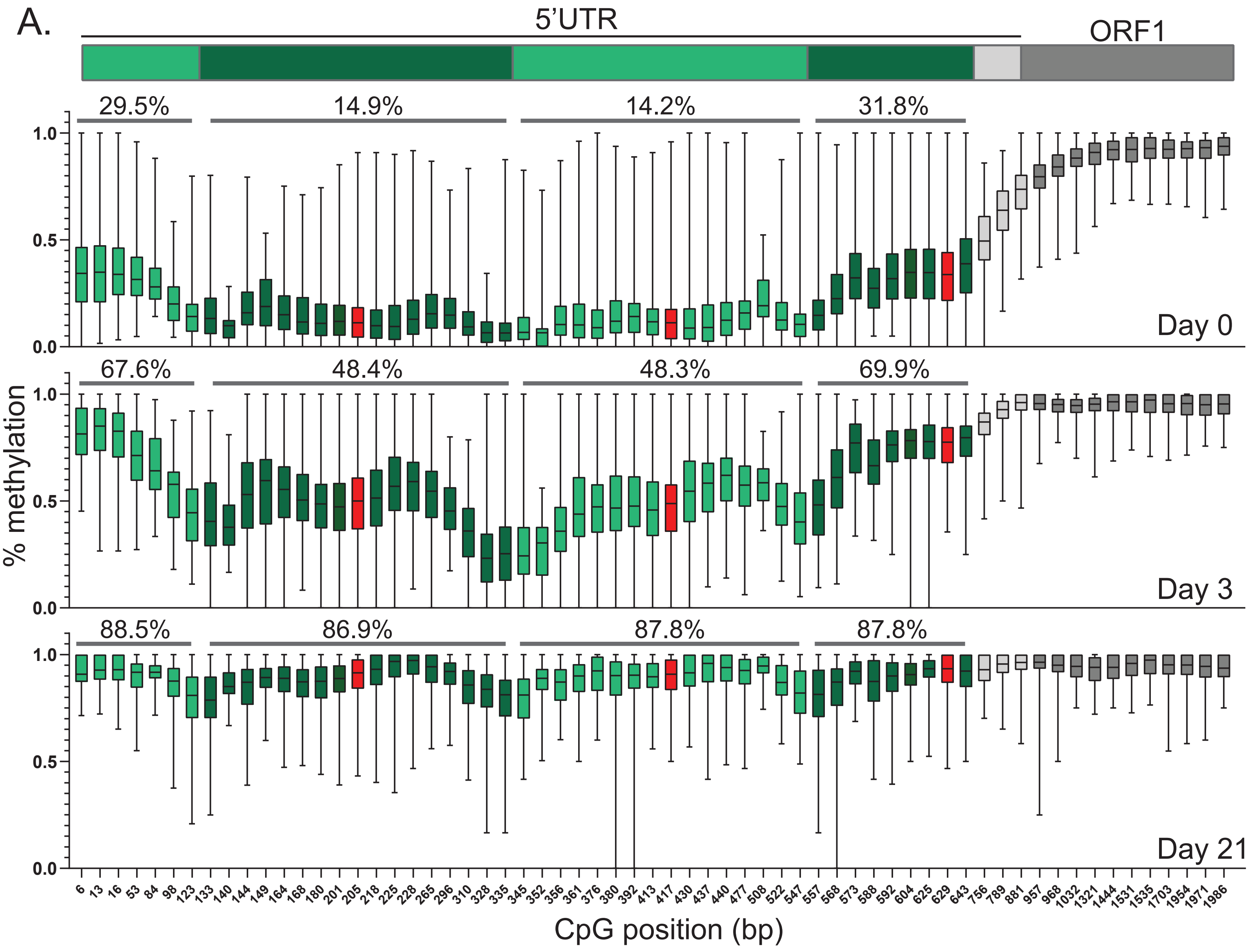

Supplemental Figure S9

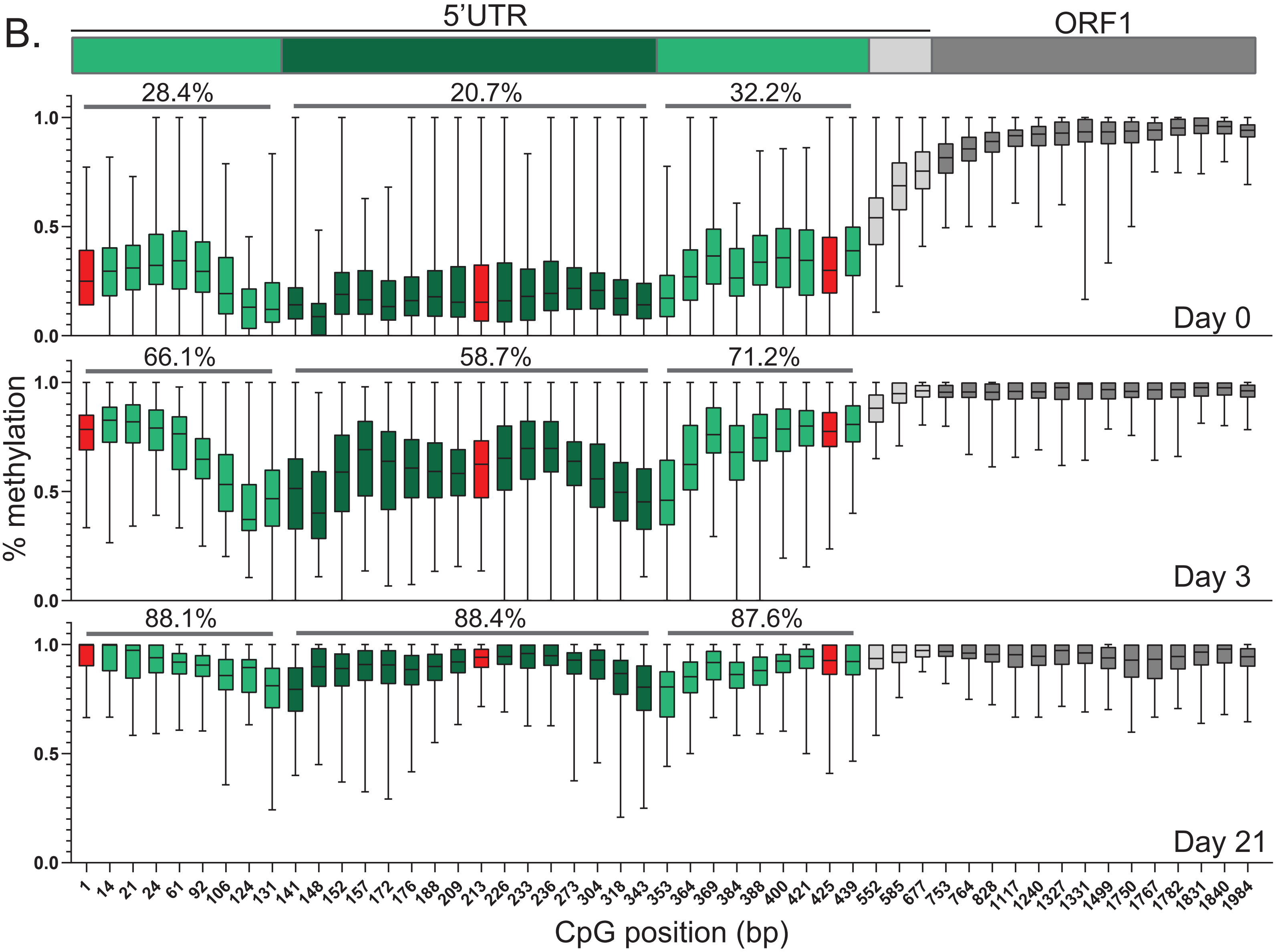

Supplemental Figure S9

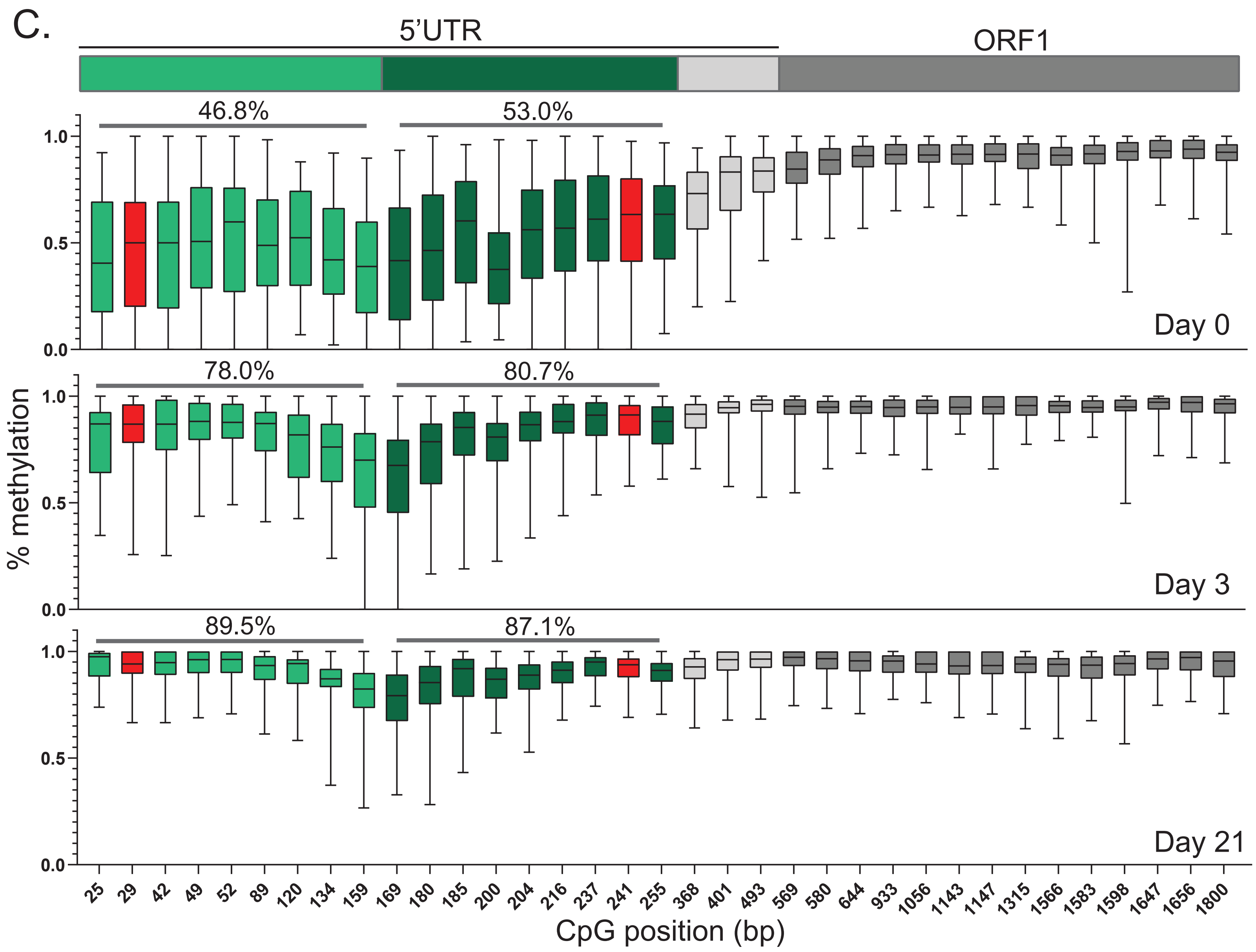

Supplemental Figure S9. Methylation of individual CpG dinucleotides across the L1 T_F promoter.

(A) Box-and-whisker plots display the median percent methylation determined by ONT sequencing for individual CpG dinucleotides across the L1 TF promoter for at least 20 individual L1 loci containing 4 monomer units, at Day 0 (top), Day 3 (middle) and Day 21 (bottom) of differentiation. The CpG positions along the X-axis are derived from a representative 4 monomer L1 T_F sequence used in our analysis (Supplemental Table S2). The central line represents median percent CpG methylation; box indicates interquartile range. Whiskers represent the top and bottom quartiles. Alternating green shading indicates CpGs belonging to each monomer unit, corresponding to the schematic of the $L1_{TF}$ 5′ UTR, above. The CpG dinucleotide partially encompassed by the core YY1 binding site is shown in red. Above each monomer unit for each box plot is shown the average percent methylation among all ≥20 L1 loci across all CpGs present within the monomer unit.

(B) As in *(A)*, but for L1 T_F loci containing 3 monomer units.

(C) As in *(A)*, but for L1 T_F loci containing 2 monomer units.
