# Supplementary material for: Locus-resolution analysis of L1 regulation and retrotransposition potential in mouse embryonic development
Source: Genome Res. 2023 Sep;33(9):1465–81. doi: 10.1101/gr.278003.123 (PMC10620060; doi:10.1101/gr.278003.123)
Supplement: Supplement 10 [file Supplemental_Fig_S10.pdf]

Supplemental Figure S10

A. Initiator Dinucleotide for all $L1T_F$ TSSs

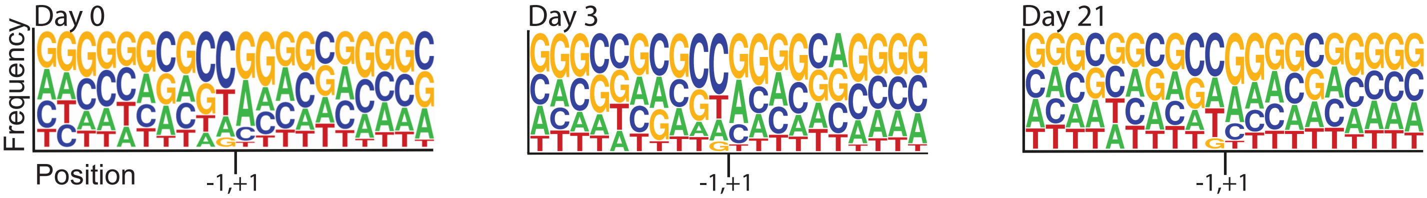

B. Initiator Dinucleotide for all $L1T_F$ TSSs

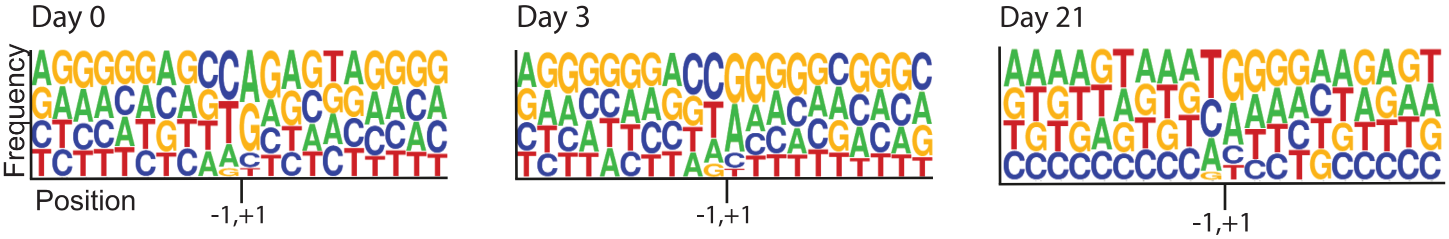

Supplemental Figure S10. Putative initiator dinucleotides inferred from L1 5′ RACE products

(A) Initiator dinucleotides for all L1 T_F TSSs, at day 0, day 3, and day 21 of differentiation.

(B) Initiator dinucleotides for the subset of TSSs originating upstream of the L1 T_F promoter region, at day 0, day 3, and day 21 of differentiation.
