# Supplementary material for: Locus-resolution analysis of L1 regulation and retrotransposition potential in mouse embryonic development
Source: Genome Res. 2023 Sep;33(9):1465–81. doi: 10.1101/gr.278003.123 (PMC10620060; doi:10.1101/gr.278003.123)
Supplement: Supplement 11 [file Supplemental_Fig_S11.pdf]

Supplemental Figure S11A

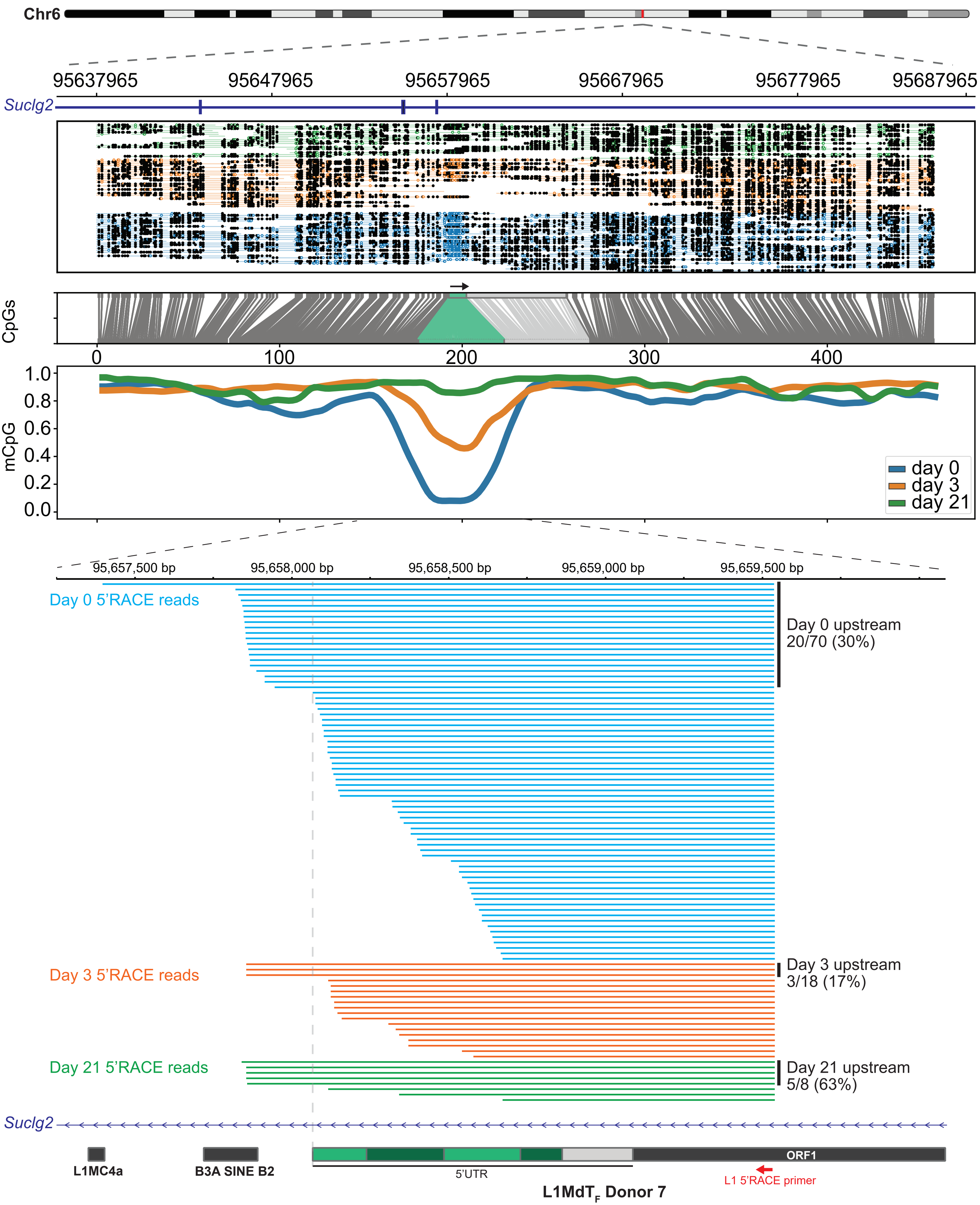

Supplemental Figure S11B

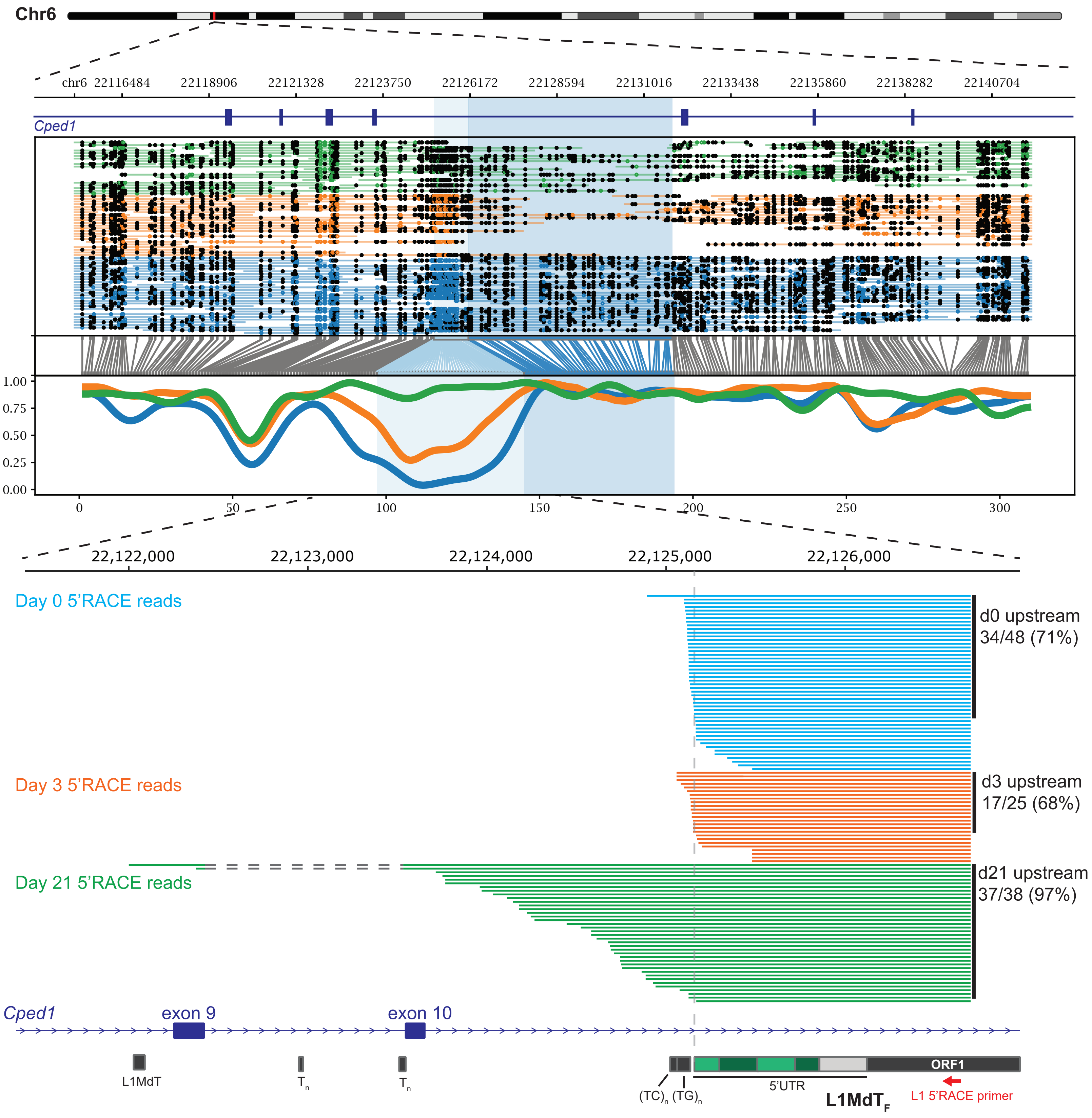

Supplemental Figure S11. Example L1 loci with upstream initiating 5'RACE reads

(A) Above, the methylation plot for Donor 7 and flanking regions (also shown in Supplemental Figure S7B). The L1 5'UTR is highlighted in green and the L1 body in gray. The position of the L1 within an intron of the *Suclg2* gene is indicated. Below, 5′ RACE reads are shown on a diagram of the 5′ end of L1 T_F element Donor 7 and flanking genomic region. A schematic of the Donor 7 element is shown, with 5′ UTR monomers denoted in alternating shades of green, and the non-monomeric 5′ UTR region shown in grey. ORF1 is shown in dark grey, and the position of the L1 5′ RACE primer is illustrated with a red arrow. The positions of two upstream TEs, an L1MC4a element and a B3A SINE B2 element are shown. 5′ RACE reads at d0 are shown in blue, d3 in orange, and d21 in green. At right, black bars highlight the upstream initiating reads at each timepoint, and the percentage and absolute count of upstream initiating reads are shown.

(B) Above, the methylation plot for an example full-length L1 T_F element with open reading frames (Chr6:22125162-22131805) and flanking genomic sequence. The L1 5'UTR is highlighted in light blue and the L1 body in dark blue. The position of the L1 within an intron of the *Cped1* gene is indicated. Below, 5′ RACE reads are shown on a diagram of the 5′ end of the L1 T_F element and 5' flanking genomic region. A schematic of the element is shown, with 5′ UTR monomers denoted in alternating shades of green, and the non-monomeric 5′ UTR region shown in grey. ORF1 is shown in dark grey, and the position of the L1 5′ RACE primer is illustrated with a red arrow. The positions of an upstream truncated L1T_F element and simple repeats are shown. 5′ RACE reads at d0 are shown in blue, d3 in orange, and d21 in green. At right, black bars highlight the upstream initiating reads at each timepoint, and the percentage and absolute count of upstream initiating reads are shown.
