# Supplementary material for: Locus-resolution analysis of L1 regulation and retrotransposition potential in mouse embryonic development
Source: Genome Res. 2023 Sep;33(9):1465–81. doi: 10.1101/gr.278003.123 (PMC10620060; doi:10.1101/gr.278003.123)
Supplement: Supplement 12 [file Supplemental_Fig_S12.pdf]

Supplemental Figure S12

A.

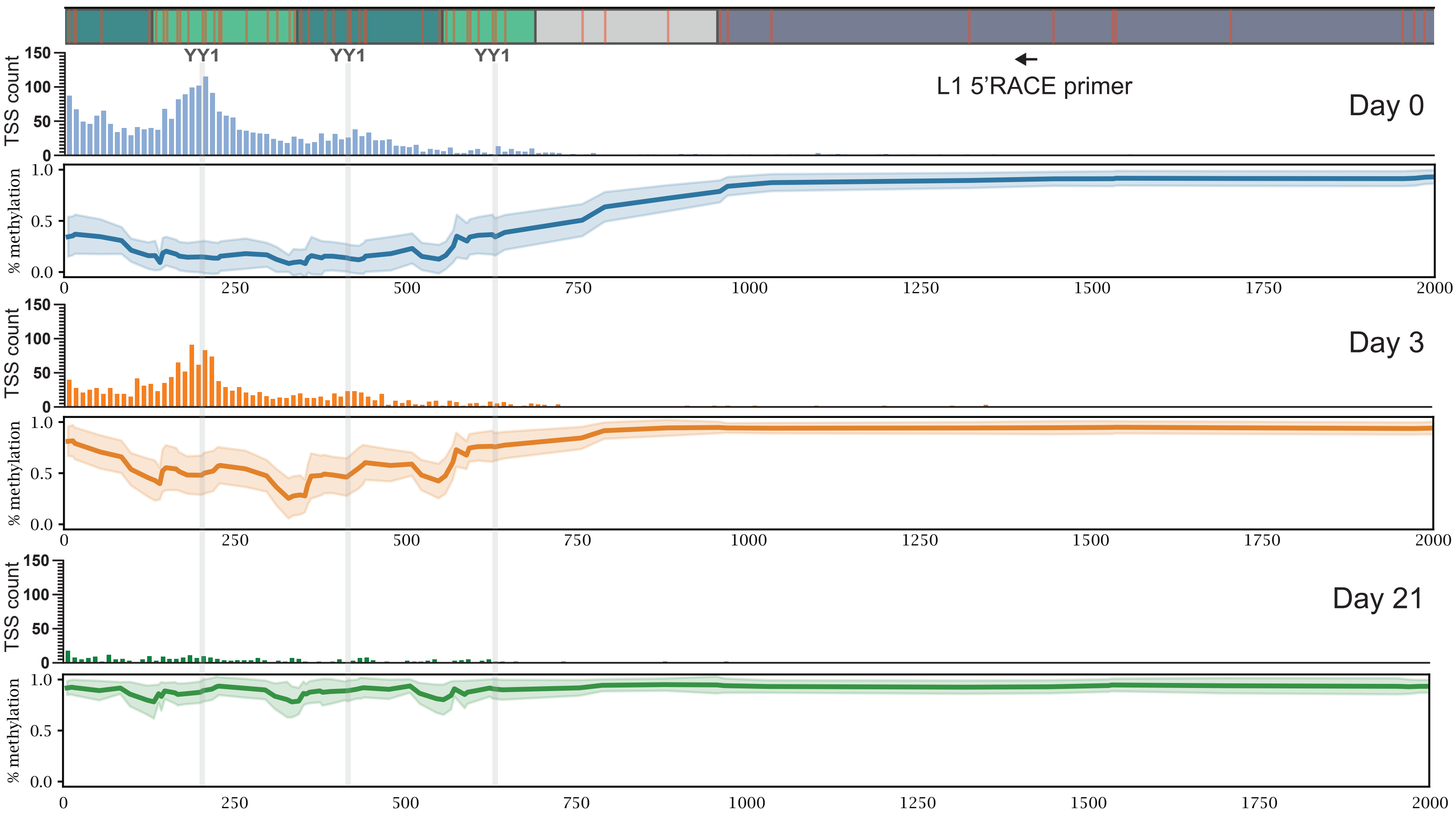

B.

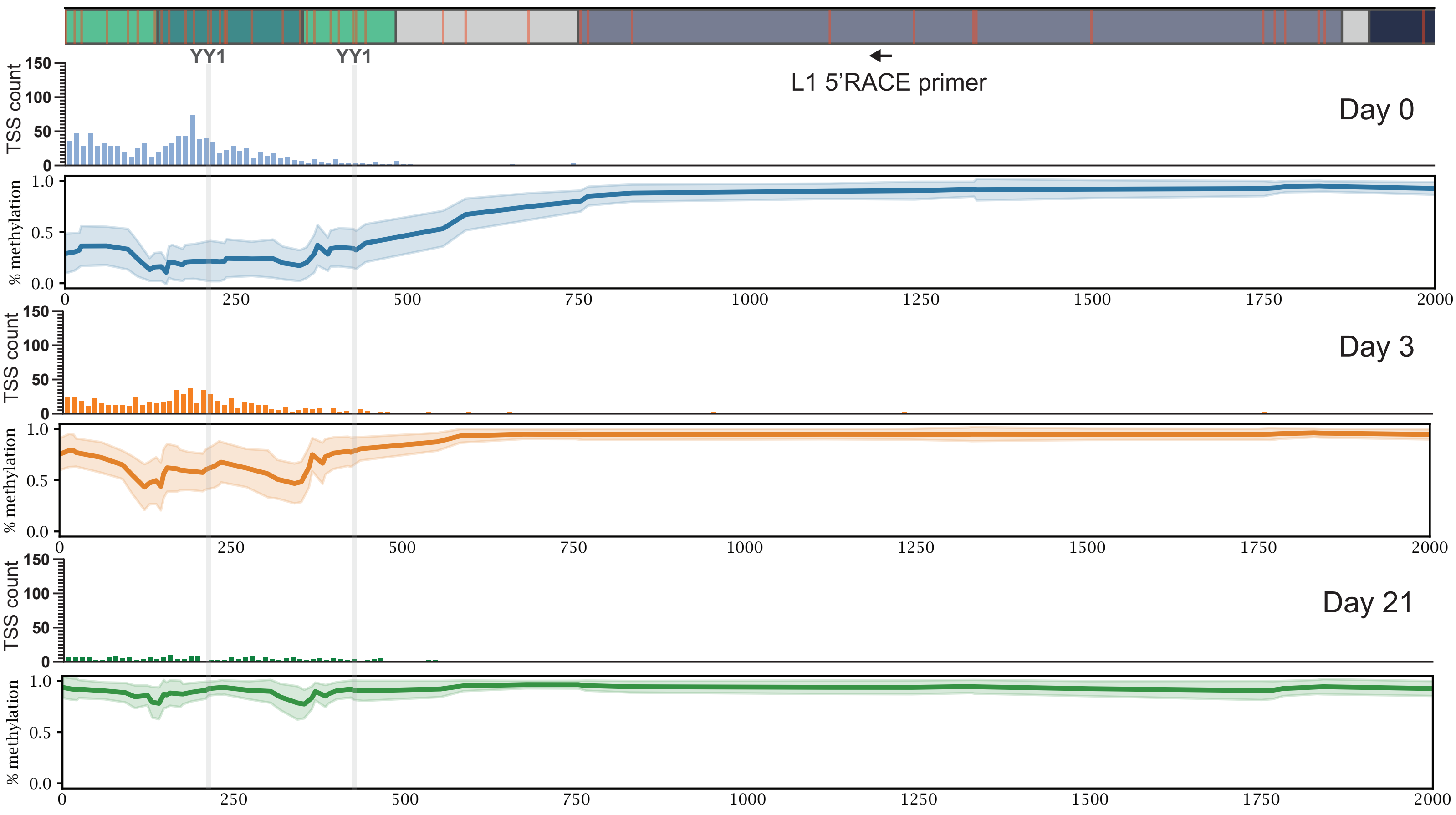

Supplemental Figure S12 (cont'd)

C.

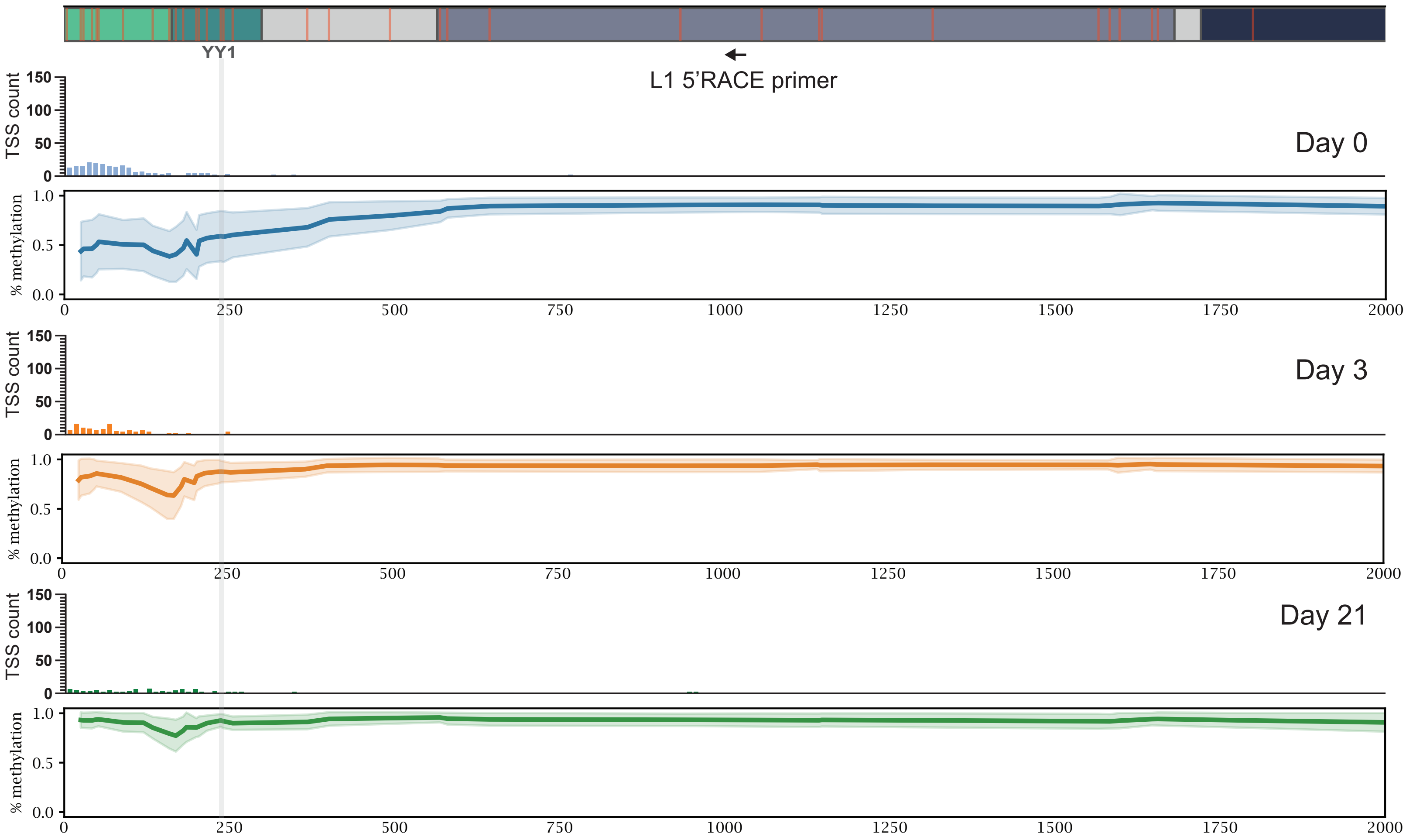

Supplemental Figure S12. Transcription start sites compared to mean DNA methylation for L1 T_{FI} elements with 2, 3, and 4 monomer units.

(A) Above, schematic of the first 2,000 bp of an L1 T_F element containing 4 monomer units. Alternating green shading represents monomer units. Light grey shading represents the non-monomeric region of the L1 TF promoter. Dark grey represents ORF1. Orange lines show the position of CpG dinucleotides. The positions of the YY1 binding sites are labelled and represented as vertical light grey lines extending down the figure panel. The position of the L1-specific 5′ RACE primer is indicated. Below, TSS counts and mean CpG methylation are shown for time points d0 (blue), d3 (orange) and d21 (green). The histograms in the upper plots show TSS count for 213 L1 T_F elements with 4 monomer units, with each bar representing a 10 bp bin. The lower plots display composite DNA methylation profiles with mean (thick line) and standard deviation (shaded region) indicated.

(B) As in *(A)* but for 229 L1 T_{FI} loci containing 3 monomer units.

(C) As in *(A)* but for 221 L1 T_{FI} loci containing 2 monomer units.
