# Supplementary material for: Locus-resolution analysis of L1 regulation and retrotransposition potential in mouse embryonic development
Source: Genome Res. 2023 Sep;33(9):1465–81. doi: 10.1101/gr.278003.123 (PMC10620060; doi:10.1101/gr.278003.123)
Supplement: Supplement 14 [file Supplemental_Methods.docx]

**Supplemental Methods**

**Mouse embryonic stem cell culture**

E14Tg2a mESCs (ATCC CRL-1821) were cultured on gelatinized tissue culture plates. Plates were coated with 0.1% gelatin (Merck, #ES-006-B) and incubated at 37°C and 5% CO_2_ for 30 min before plating. Cells were passaged at 70-80% confluence every 2-3 days using Trypsin 0.25% EDTA (Thermo Fisher Scientific). mESCs were plated at a density of 1 × 10^5^ cells/ml. Medium was changed every day for optimal growth. mESCs for the retrotransposition assays were cultured in 2i+serum conditions in 1:1 DMEM/F12 media:neurobasal media (Thermo Fisher Scientific) supplemented with 0.5 % N2 and 0.5% B27 (Thermo Fisher Scientific), 10% FBS (Thermo Fisher Scientific, batch tested), 1% L-glutamine (Thermo Fisher Scientific), 1% penicillin-streptomycin (Thermo Fisher Scientific), 0.1 mM β-mercaptoethanol (Sigma-Aldrich, #M3148), 1 μM PD0325901 (Sigma-Aldrich, #PZ0162) and 3 μM CHIR99021 (Sigma-Aldrich, #SML1046). mESCs for methylation analysis were cultured in 2i+serum conditions as above, 2i+LIF conditions in 1:1 DMEM/F12 media:neurobasal media (Thermo Fisher Scientific) supplemented with N2 and B27 (Thermo Fisher Scientific), 1% L-glutamine (Thermo Fisher Scientific), 1% penicillin-streptomycin (Thermo Fisher Scientific), 0.1% β-mercaptoethanol (Sigma-Aldrich, #M3148), 1 μM PD0325901 (Sigma-Aldrich, #PZ0162), 3 μM CHIR99021 (Sigma-Aldrich, #SML1046) and 1000U/ml ESGRO mLIF (Merck, #ESG1107) and serum+LIF conditions in Knockout DMEM supplemented with 10% FBS (Thermo Fisher Scientific, batch tested), 1% non-essential amino acids (NEAA) (Thermo Fisher Scientific), 1% L-glutamine (Thermo Fisher Scientific), 1% penicillin-streptomycin (Thermo Fisher Scientific), 0.1 mM β-mercaptoethanol (Sigma-Aldrich, #M3148) and 1000U/ml ESGRO mLIF (Merck, #ESG1107).

**mESC differentiation**

E14Tg2a mESCs (ATCC CRL-1821) were differentiated into embryoid bodies (EBs) as previously described (Behringer et al. 2016). Hanging drop culture was used to generate EBs of comparable dimension. Bacteriological-grade 10 cm plates were prepared by adding 15 ml 1× PBS (Invitrogen) to each plate and incubating them at 37°C and 5% CO_2_. mESCs cultured in serum+LIF conditions were harvested at 60-70% confluency by adding Trypsin 0.25% EDTA (Thermo Fisher Scientific). The trypsinized cells were resuspended in EB medium (Knockout DMEM (Thermo Fisher Scientific, 10% FBS (Thermo Fisher Scientific, batch tested), 1% non-essential amino acids (NEAA) (Thermo Fisher Scientific), 1% L-glutamine (Thermo Fisher Scientific), 1% penicillin-streptomycin (Thermo Fisher Scientific), 0.1% β-mercaptoethanol (Sigma-Aldrich, #M3148)) and centrifuged at 400 ×g for 5 mins. Supernatant was removed and cells were resuspended in EB medium. The cell concentration was determined using the TC20 Automated Cell Counter (Bio-Rad) and the cell suspension was diluted to 3 × 10^4^ cells/ml. 30 μl cell suspension drops (1000 cells/drop) were distributed inside the lid of the previously prepared bacteriological-grade 10 cm plates. The lid was carefully placed back on the base containing 1× PBS (Invitrogen). The EBs were incubated for three days. On day three, EBs were collected from lids and transferred to six-well ultra-low adherence plates (Corning). 2 ml EB media were added to each well and the EBs were incubated for another three days. Six day old EBs were plated onto six-well plates coated with 0.1% gelatin (Merck) in EB medium. After 24 h, the EB medium was replaced by differentiation medium (Knockout DMEM (Thermo Fisher Scientific), 1% non-essential amino acids (NEAA) (Thermo Fisher Scientific), 1% L-glutamine (Thermo Fisher Scientific), 1% penicillin-streptomycin (Thermo Fisher Scientific), 0.1% β-mercaptoethanol (Sigma-Aldrich, #M3148)). The cells were differentiated for two weeks. Medium was changed every other day and cells for DNA extraction were collected every three days.

**Immunostaining**

Immunostaining was performed on day 7 (24 h after plating of EBs) and day 21 of mESC differentiation. EBs were plated on gelatinized coverslips in twelve-well plates. Coverslips harboring cells were rinsed with 1× PBS (Invitrogen) and fixed in 4% paraformaldehyde in PBS for 20 mins at room temperature. Coverslips were washed twice with 1× PBS for 5 min followed by 1 min incubation in permeabilization buffer PBT (1x PBS, 0.5% Triton X-100 (Sigma-Aldrich)). Coverslips were washed in 1×PBS for 10 min and blocked in PBT containing 10% normal donkey serum (blocking buffer) for 1 h at room temperature. Primary antibodies (β-tubulin, Rabbit IgG (Sigma-Aldrich, #T2200), 1:500; Α-fetoprotein (AFP), Goat IgG (R&D Systems, #AF5369), 1:200; Smooth muscle actin, Mouse IgG (Thermo Fisher Scientific, #14976080), 1:500) were diluted in blocking buffer and incubated for 1 h at room temperature, then washed with 1× PBS. Secondary antibodies (Alexa Fluor 647 Donkey Anti-Rabbit IgG (Jackson ImmunoResearch, #711-606-152), 1:500; Cy3 Donkey Anti-Goat IgG (Jackson ImmunoResearch, #715-165-150), 1:200; Alexa Fluor 488 Donkey Anti-Mouse IgG (Jackson ImmunoResearch, #715-546-150), 1:500) were diluted in blocking buffer and incubated for 1 h at room temperature, then washed with 1× PBS. Cells were stained with H33258 (1:1000 in 1× PBS) for 5 min and washed with 1× PBS for 10 min. PermaFluor mounting media (Thermo Fisher Scientific, #TA-030-FM) was added to glass chamber slides and coverslips were carefully placed to avoid the creation of bubbles and dried overnight at room temperature. Cells were imaged on a spinning-disk confocal system (Marianas; 3I, Inc.) consisting of an Axio Observer Z1 (Carl Zeiss) equipped with a CSU-W1 spinning-disk head (Yokogawa Corporation of America), ORCA-Flash4.0 v2 sCMOS camera (Hamamatsu Photonics) and a 20× 0.8 NA PlanApo objective. Image acquisition was performed using SlideBook 6.0 (3I, Inc). Image processing and analysis was done using Image J 1.52 software.

**Generation of mouse L1 reporter constructs**

DNA sequences corresponding to the different donor/daughter mouse L1s were amplified from genomic DNA using Expand Long Range dNTPack (Roche). Reaction mixes contained 5 μl 5× Expand Long Range Buffer with 12.5 mM MgCl_2_, 1.25 μl dNTP Mix (dATP, dCTP, dGTP, dTTP at 10 mM each), 1.25 μl DMSO (100%), 1 μl primer mix (50 μM of each primer), 0.35 μl Expand Long Range Enzyme Mix (5 U/μl), 4-10 ng genomic DNA template and molecular grade water up to a total volume of 25 μl. PCRs were performed with the following cycling conditions: 92˚C for 3 min, 10 cycles of 92˚C for 30 sec, 56-60˚C for 30 sec, and 68˚C for 5-7.5 min; 25 cycles of 92˚C for 30 sec, 56-60˚C for 30 sec, and 68˚C for 5-7 min plus 20 sec/cycle elongation for each successive cycle, followed by 68˚C for 10 min. Primers (Supplemental Table S1) introduced a *NotI* restriction site at the L1 5ʹ end. Full-length L1 elements were then cloned into pGEMT Easy Vector (Promega) according to the manufacturer’s instructions. Ligations were incubated overnight at 4˚C. Ligation reactions were transformed using One Shot TOP10 chemically competent *E. coli* (Invitrogen) according to the manufacturer’s instructions. Blue/white screening was performed using LB/ampicillin/IPTG/X-Gal plates. 3-5 positive colonies per L1 element were chosen for Miniprep culture and plasmid DNA was isolated using QIAprep Spin Miniprep Kit (Qiagen) according to the manufacturer’s instructions. At least three clones per element were capillary sequenced using L1 sequencing primers (Supplemental Table S1). Sequences from at least three clones of the same L1 element were compared to each other to identify PCR-induced mutations. L1s were then reconstructed by combination of non-mutated fragments from different clones using restriction enzymes (New England Biolabs) cutting within the L1 sequence.

The L1 3ʹend fragment was produced by PCR amplification from plasmid DNA using a forward primer upstream of a *HindIII*-site in ORF2 and a reverse primer introducing an *SbfI*-site at the end of the L1 sequence thereby removing the polyadenylation signal (AATAAA) and the G-rich region (GRR). The GRR was amplified using primers that introduce an *AgeI*- and *PacI*-site on each site of the GRR and removing the polyadenylation signal. All restriction enzymes used for cloning were obtained from New England Biolabs. Restriction digests were performed according to the manufacturer’s instructions (New England Biolabs). Reactions were purified using agarose gel electrophoresis and target fragments were excised and purified using QIAquick and MinElute Gel Extraction Kits (Qiagen) according to the manufacturer’s protocol.

A modified version of the previously described pTN201 construct (Naas et al. 1998) in which the L1 3ʹ UTR GRR is located downstream, rather than upstream, of the NEO indicator cassette was used as a backbone to generate L1 reporter constructs (Richardson et al. 2022). L1spa was removed from the pCEP4 backbone using *NotI* and *SbfI*. The pCEP4 backbone was dephosphorylated using Calf Intestinal Alkaline Phosphatase (CIP) (New England Biolabs) according to the manufacturer’s instructions. The backbone and multiple fragments from the target L1 (Insertion 2, Insertion 5, Insertion 7, polyL1Tf_3, polyL1Tf_4, Donor 2, Donor 5, Donor 7, Donor 3, Donor 4) were combined in a single ligation reaction using T4 DNA Ligase (New England Biolabs) according to the manufacturer’s instructions and incubated overnight at 16˚C. Ligations were transformed using One Shot TOP10 chemically competent *E. coli* (Invitrogen) according to the manufacturer’s instructions. Plasmid DNA of positive clones was obtained using QIAprep Spin Miniprep Kit (Qiagen). The absence of mutations was verified by capillary sequencing. The GRR of L1spa was replaced by the respective GRR of each L1 element using *AgeI* and *PacI*. The backbone and GRR were ligated and transformed as described above. Plasmid DNA for the retrotransposition assays was obtained using Plasmid Maxi kit (Qiagen). The absence of mutations was verified by capillary sequencing. Each construct was built with and without a CMV promoter upstream of the L1.

Insertion 2 has a C to T mutation in the YY1 binding motif in its first monomer upstream of the unique region. To fix the mutation, a reverse primer (Insertion_2_YY1_fix_R) overlapping the mutation with the correct YY1 binding motif sequence was designed (Supplemental Table S1). The promoter was then amplified from the construct prepared for the retrotransposition assay using Q5 High-Fidelity 2× Master Mix (New England Biolabs). Primers and annealing temperatures are listed in Supplemental Table S1. Reaction mixes contained 12.5 μl Q5 High-Fidelity 2× Master Mix, 1.25 μl primer mix (10 μM of each primer), 2 ng plasmid DNA template and molecular grade water up to a total volume of 25 μl. PCRs were performed using the following conditions: 98˚C for 2 min, 35 cycles of 98˚C for 10 sec, 62-72˚C for 30 sec, and 72˚C for 1 min, followed by 72˚C for 2 min. Insertion_2_FL_F was used as a forward primer. The PCR fragment was then digested with *NotI* and *XmaI*. pCEP4ΔCMV-mneoI-G4-Ins2 was used as a backbone and digested with *NotI* and *SbfI*. The backbone was dephosphorylated using Calf Intestinal Alkaline Phosphatase (CIP) (New England Biolabs) according to the manufacturer’s instructions. The rest of the Insertion 2 element was obtained by digesting pCEP4ΔCMV-mneoI-G4-Ins2 with *XmaI* and *SbfI*. The three fragments were then ligated and transformed as described above. Plasmid DNA of positive clones was obtained using Plasmid Maxi Kit (Qiagen). The absence of mutations was verified by capillary sequencing.

**Retrotransposition assay**

Retrotransposition assays in HeLa-JVM cells were performed as previously described (Kopera et al. 2016) with some minor modifications. HeLa-JVM cells were grown in DMEM complete medium. To assay L1 retrotransposition of L1 donor/daughter pairs, HeLa-JVM were seeded at a density of 5×10^3^ cells/well in six-well tissue culture plates. To assay Insertion 2-YY1-fixed, HeLa-JVM were seeded at a density of 1×10^4^ cells/well. 14-16 h after plating, cells were transfected with L1 reporter constructs using 4 μl FuGENE HD transfection reagent (Promega), 96 μl Opti-MEM (Thermo Fisher Scientific) and 1 μg plasmid DNA per well. Transfection efficiency was determined in parallel by preparing transfection mixes containing 4 μl FuGENE HD transfection reagent (Promega), 96 μl Opti-MEM (Thermo Fisher Scientific), 0.5 μg L1 expression plasmid and 0.5 μg pCEP4-eGFP. 100 μl transfection mixture was added to each well containing 2 mL DMEM complete medium. The plates were incubated at 37˚C and 5% CO_2_. The transfection was stopped by replacing the medium 24 h post-transfection. Transfection efficiency was determined 72 h post-transfection. pCEP4-eGFP co-transfected wells were trypsinized and cells were collected from each well and centrifuged at 400 ×g for 5 min. Cell pellets were resuspended in 300-500 μl 1× PBS (Invitrogen). 10 μl Propidium iodide (Thermo Fisher Scientific) were added to cell suspensions. The number of EGFP-positive cells was determined using a CytoFLEX flow cytometer (Beckman Coulter). The percentage of EGFP-positive cells was used to normalize the G418-resistant colony counts for each L1 reporter construct (Kopera et al. 2016). Geneticin/G418 (400 μg/ml) (Thermo Fisher Scientific) selection was started 3 days post-transfection and performed for 12 days. G418-resistant foci were washed with 1× PBS and fixed using 2% Formaldehyde/0.2% Glutaraldehyde in 1× PBS (Sigma-Aldrich) fixing solution at room temperature for 30 min. Staining was done using 0.1% Crystal Violet solution (Sigma-Aldrich) at room temperature for 10 min. Foci were counted in each well. Three biological replicates, each containing three technical replicates per L1 construct were done.

**Supplemental references**

Behringer R, Gertsenstein M, Nagy KV, Nagy A. 2016. Differentiating mouse embryonic stem cells into embryoid bodies by hanging-drop cultures. *Cold Spring Harb Protoc* **2016**: 1073–1076.

Kopera HC, Larson PA, Moldovan JB, Richardson SR, Liu Y, Moran J V. 2016. Line-1 cultured cell retrotransposition assay. In *Methods in Molecular Biology*, Vol. 1400 of, pp. 139–156, Humana Press Inc.

Naas TP, DeBerardinis RJ, Moran J V, Ostertag EM, Kingsmore SF, Seldin MF, Hayashizaki Y, Martin SL, Kazazian HH. 1998. An actively retrotransposing, novel subfamily of mouse L1 elements. *EMBO J* **17**: 590–597.

Richardson SR, Chan D, Gerdes P, Han JS, Boeke JD, Faulkner GJ. 2022. Revisiting the impact of synthetic ORF sequences on engineered LINE-1 retrotransposition. *bioRxiv* 2022.08.29.505632.
